# Dual regulation of the actin cytoskeleton by CARMIL-GAP

**DOI:** 10.1242/jcs.258704

**Published:** 2022-06-20

**Authors:** Goeh Jung, Miao Pan, Christopher J. Alexander, Tian Jin, John A. Hammer

**Affiliations:** 1 Cell and Developmental Biology Center, National Heart, Lung, and Blood Institute, National Institutes of Health, Bethesda, MD 20892, USA; 2 Chemotaxis Signal Section, Laboratory of Immunogenetics, National Institute of Allergy and Infectious Disease, National Institutes of Health, Rockville, MD 20852, USA

**Keywords:** CARMIL, Capping protein, Rho GTPase, GAP domain, Phagocytosis

## Abstract

Capping protein Arp2/3 myosin I linker (CARMIL) proteins are multi-domain scaffold proteins that regulate actin dynamics by regulating the activity of capping protein (CP). Here, we characterize CARMIL-GAP (GAP for GTPase-activating protein), a *Dictyostelium* CARMIL isoform that contains a ∼130 residue insert that, by homology, confers GTPase-activating properties for Rho-related GTPases. Consistent with this idea, this GAP domain binds *Dictyostelium* Rac1a and accelerates its rate of GTP hydrolysis. CARMIL-GAP concentrates with F-actin in phagocytic cups and at the leading edge of chemotaxing cells, and CARMIL-GAP-null cells exhibit pronounced defects in phagocytosis and chemotactic streaming. Importantly, these defects are fully rescued by expressing GFP-tagged CARMIL-GAP in CARMIL-GAP-null cells. Finally, rescue with versions of CARMIL-GAP that lack either GAP activity or the ability to regulate CP show that, although both activities contribute significantly to CARMIL-GAP function, the GAP activity plays the bigger role. Together, our results add to the growing evidence that CARMIL proteins influence actin dynamics by regulating signaling molecules as well as CP, and that the continuous cycling of the nucleotide state of Rho GTPases is often required to drive Rho-dependent biological processes.

## Abstract (precis)

**Summary:** *Dictyostelium* CARMIL-GAP supports phagocytosis and chemotaxis by regulating both capping protein and Rac1.

## INTRODUCTION

*Dictyostelium* capping protein Arp2/3 myosin I linker (CARMIL) and its ortholog Acan 125 in *Acanthamoeba* are the founding members of a class of proteins that regulate capping protein (CP), the primary actin filament barbed end capping protein in most, if not all, eukaryotic cells, and a central player in the assembly, organization and dynamics of the actin cytoskeleton (Edwards et al., 2014; Jung et al., 2001; Xu et al., 1995). Identified based on their interaction with the SH3 domains of type 1 myosins (Jung et al., 2001; Xu et al., 1995), these ∼1100-residue, multidomain scaffold proteins and their metazoan counterparts contain a ∼50-residue domain that binds CP with nanomolar affinity (Remmert et al., 2004; Uruno et al., 2006; Yang et al., 2005). This domain, referred to in the literature as either CARMIL homology domain 3 (CAH3) or capping protein interacting domain (CPI), exerts two dramatic and interrelated biochemical effects on CP. First, when bound to CP, CPI reduces the affinity of CP for the fast-growing barbed end of the actin filament from 0.1 nM to ∼30 nM (Uruno et al., 2006; Yang et al., 2005). This reduction in affinity equates to a reduction in the half-life of CP on the barbed end from ∼30 min to ∼10 s. Second, when added to CP-capped actin filaments, CPI dramatically accelerates the dissociation of CP from the barbed end such that, at saturation, CPI reduces the half-life of CP on the barbed end from ∼30 min to ∼10 s (Fujiwara et al., 2010). Importantly, these two biochemical effects are variations of the same underlying mechanism, namely, that the binding of CPI to CP alters the conformation of CP in such a way as to reduce its affinity for the barbed end by several hundred fold (Hernandez-Valladares et al., 2010; Kim et al., 2012; Takeda et al., 2010; Zwolak et al., 2010b). Stated another way, the allosteric effect of CPI on CP serves to both reduce the affinity of preformed CP–CPI complexes for the barbed end, and promote uncapping when CPI is added to filaments already capped with CP.

Although the biochemical effects exerted by CPI on CP *in vitro* would suggest that CARMIL proteins function as CP antagonists *in vivo*, the truth may well be the opposite. To understand how this is possible, one must consider CARMIL function in cells in the context of a second direct regulator of CP, known as V-1 (also known as myotrophin in mammals). This ubiquitously expressed, small ankyrin repeat protein binds CP in a 1:1 ratio with ∼20 nM affinity to prevent it from capping the barbed end (Bhattacharya et al., 2006; Takeda et al., 2010; Zwolak et al., 2010a). Importantly, V-1 is present in the cytoplasm at a 3- to 4-fold molar excess over CP (Fujiwara et al., 2014; Jung et al., 2016). Given this, and given the affinity of V-1 for CP, one would predict that ∼99% of cellular CP would be sequestered by V-1, barring regulation. Critically, CARMIL's CPI domain provides a powerful counter to V-1's sequestering activity by robustly catalyzing an exchange reaction that converts CP:V-1 complexes into CP:CPI complexes (Fujiwara et al., 2014; Johnson et al., 2018; Takeda et al., 2010). In this way, CARMIL proteins convert inactive CP (CP–V-1) into a version (CP–CPI) with moderate (i.e. ∼30 nM) affinity for the barbed end. In other words, in the face of pervasive sequestration of CP by V-1, CARMIL proteins would be expected to serve as activators rather than inhibitors of CP. These findings, when combined with other data regarding the localization and activation of CARMIL at the plasma membrane (Fujiwara et al., 2014; Zwolak et al., 2013), argue that CARMIL and V-1 cooperate at the leading edge of cells to promote Arp2/3 complex-dependent branched actin network assembly there by promoting weak barbed-end capping (Fujiwara et al., 2014). Consistent with this model, estimates of the half-life of CP on barbed ends near the plasma membrane *in vivo* (∼2 to 15 s) (Iwasa and Mullins, 2007; Miyoshi et al., 2006) are much closer to the half-life of the CP–CPI complex on the barbed end (∼10 s) than to the half-life of CP alone (∼30 min) (Fujiwara et al., 2010). This model is also consistent with evidence that CARMIL proteins promote lamellipodia formation (Edwards et al., 2015; Jung et al., 2001; Liang et al., 2013), that cells forced to express a version of CP that can cap barbed ends but cannot ‘see’ the CPI motif exhibit a CP-knockdown phenotype (Edwards et al., 2015), and that the defects in actin organization and dynamics exhibited by cells devoid of V-1 or overexpressing V-1 both demonstrate that V-1 regulates CP activity *in vivo* (Jung et al., 2016).

Although this brief overview of the CP1 domain of CARMIL emphasizes the role played by CARMIL proteins in regulating actin assembly by regulating CP activity, there is growing evidence that these scaffold proteins also regulate actin assembly by regulating signaling pathways (reviewed in Stark et al., 2017). One clear example is the *C. elegans* CARMIL homolog CRML-1, which negatively regulates neuronal growth cone migration by binding to and inhibiting UNC-73, the *C. elegans* homolog of Trio, a guanine nucleotide exchange factor (GEF) for Rac and Rho (Vanderzalm et al., 2009). Consistent with this finding, immunoprecipitates of CARMIL-1 from human fibroblasts contain Trio (Liang et al., 2009), and the CARMIL-2 interactome in T cells contains two GEFs for Rho-related GTPases (VAV1 and DOCK8) (Liang et al., 2013). Interestingly, CARMIL-2 also serves to link the cell surface receptor CD28 in T cells to the adaptor molecule CARMA1, which then collaborates with protein kinase Cθ (PKCθ) to promote full T cell activation by activating the transcription factor NF-κB (Liang et al., 2013; Roncagalli et al., 2016).

In our 2001 study of *Dicytostelium* CARMIL, we showed that it binds myosin 1, CP and the Arp2/3 complex, and that it concentrates with them in several actin-rich structures, most notably macropinocytic projections on the dorsal surface of vegetative cells and pseudopods at the leading edge of starved aggregating cells (Jung et al., 2001). Consistent with these localizations, CARMIL-null cells exhibited pronounced defects in macropinocytosis and chemotactic aggregation (Jung et al., 2001). Whereas the CARMIL isoform examined in that study was considered at the time to be the only CARMIL isoform in *Dictyostelium*, the subsequent completion of the *Dictyostelium* genome sequence revealed the presence of a second CARMIL gene. We call this second isoform CARMIL-GAP because the protein contains an apparent GTPase-activating protein (GAP) domain for Rho-related GTPases in addition to all the normal domains present in CARMILs. Here, we characterized the localization and cellular functions of CARMIL-GAP, and we used complementation to parse out the relative contributions made by its CPI and GAP domains to its cellular functions. Together, our results add to the growing evidence that CARMIL proteins regulate actin dynamics by regulating signaling molecules as well as CP, although in the case of CARMIL-GAP this dual regulation is accomplished not in trans but by this one protein. Finally, our results support the emerging concept (Denk-Lobnig and Martin, 2019) that the continuous cycling of Rho GTPases between their GTP and GDP bound states through the coordinated action of their GEFs and GAPs is often required to drive Rho-dependent biological processes forward.

## RESULTS

### CARMIL-GAP contains functional GAP and CPI domains

**Fig. 1.**
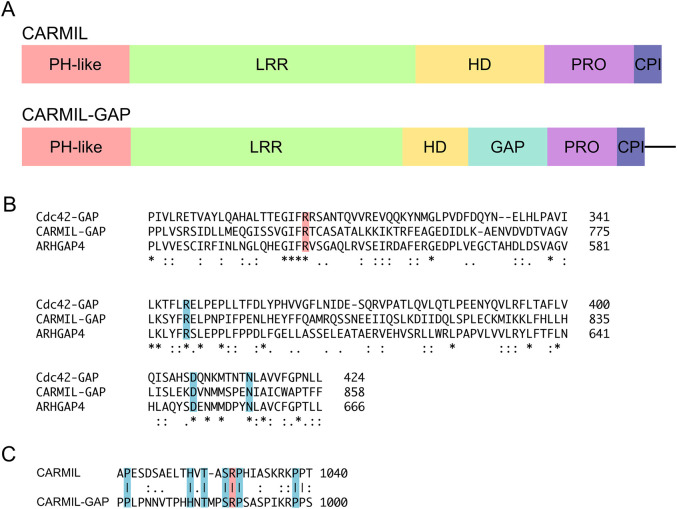
**Domain organization of CARMIL-GAP.** (A) Diagram depicting the domain organization of CARMIL-GAP versus CARMIL (Jung et al., 2001). PH-like, pleckstrin homology-like domain (Zwolak et al., 2013); LRR, leucine-rich repeat domain; HD, homodimerizing domain (Zwolak et al., 2013); Pro, proline-rich domain; CPI, capping protein interaction domain. The sequence at the C-terminus of CARMIL-GAP that is indicated in the diagram by the straight line is not present in CARMIL. (B) Alignment of the putative GAP domain of CARMIL-GAP with the GAP domains of human Cdc42-GAP (also known as ARHGAP1) (Vogt et al., 2007) and human ARHGAP4 (Barfod et al., 1993). The conserved arginine at position 737 in CARMIL-GAP, 305 in Cdc42-GAP and 544 in ARHGAP4 (shaded red) is required for robust GAP activity. The conserved residues shaded blue are required for stabilization of the switch domain of Rho-GTPases during GAP-stimulated GTP hydrolysis. (C) Alignment of the CPI domains in CARMIL and CARMIL-GAP. The arginine at position 1029 in CARMIL and 989 in CARMIL-GAP (shaded red) is essential for binding CP with high affinity (Edwards et al., 2014; Uruno et al., 2006; Yang et al., 2005). The residues shaded blue were identified by site-directed mutagenesis of the CPI domain from *Acanthamoeba* CARMIL as contributing significantly to its anti-CP activity (Uruno et al., 2006). Lines indicate identity, colons indicate highly conservative substitutions, and dots indicate moderately conservative substitutions.

Alignment of the amino acid sequence of *Dictyostelium* CARMIL-GAP with that of *Dictyostelium* CARMIL (Fig. S1) shows that CARMIL-GAP contains, in addition to the five domains found in all CARMIL proteins [a pleckstrin homology-like (PH-like) domain, a leucine rich repeat (LRR) domain, a homo-dimerizing (HD) domain, a proline-rich (Pro) domain, and a CPI domain (Edwards et al., 2013)], a ∼145-residue sequence inserted between its HD and Pro domains (Fig. 1A). Blast searches revealed that this insert is homologous to the GAP domains present in GAPs for Rho-related GTPases, such as Cdc42-GAP and ARHGAP4 (Barfod et al., 1993; Vogt et al., 2007) (Fig. 1B). Importantly, this putative GAP domain in CARML-GAP contains an arginine residue present in all GAP domains that is required for robust GAP activity (residue 737; highlighted red in Fig. 1B), as well as polar residues at positions 780, 841 and 849 (highlighted blue in Fig. 1B) that are required for stabilization of the Rho-GTPase's switch domain during GAP-stimulated GTP hydrolysis (Nassar et al., 1998; Rittinger et al., 1997; Scheffzek et al., 1997). Together, these sequence elements suggest that CARMIL-GAP functions at least in part as a GAP for a Rho-related GTPase.

**Fig. 2.**
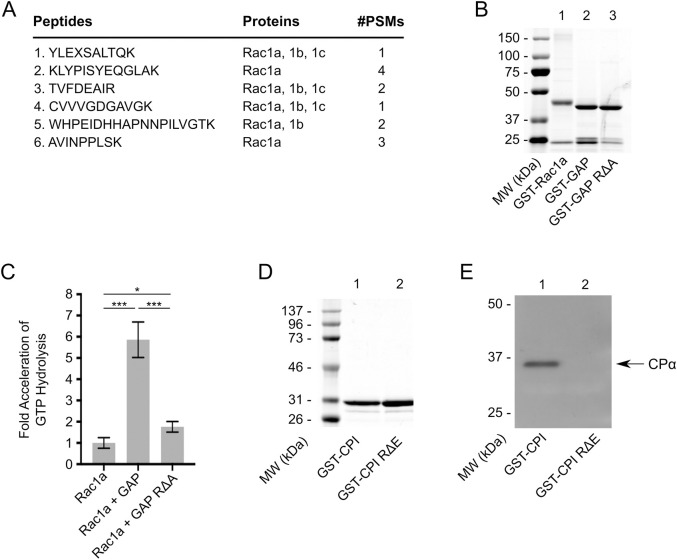
**CARMIL-GAP contains functional GAP and CPI domains.** (A) Shown are the six unique Rac1 peptides obtained by mass spectrometry analysis of the GST–GAP domain pull down, the Rac1 isoform they were found in, and the frequency with which each was identified (peptide spectrum matches, PSMs). (B) Coomassie Blue-stained gel of purified GST–Rac1a (lane 1), GST-GAP (lane 2) and GST–GAP-RΔA (lane 3). (C) Shown is the mean±s.d. fold activation of GTP hydrolysis rate of GST–Rac1a (mean normalized to 1.0) by GST–GAP or GST–GAP-RΔA (Rac1a, 1.00±0.25; Rac1a+GAP, 5.80±0.84; Rac1a+GAP RΔA, 1.74±0.25; *N*=3). (D) Coomassie Blue-stained gel of purified GST–CPI and GST–CPI-RΔA. (E) Western blot of the material eluted from GST–CPI beads (lane 1) and GST–CPI-RΔA beads (lane 2) after incubation with whole-cell extracts, and probed with an antibody to the α subunit of *Dictyostelium* CP. Gel and blot images are representative of three repeats.

To seek evidence that the putative GAP domain in CARMIL-GAP functions as a GAP, we first sought to identify the Rho-related GTPase(s) that interact with it. In terms of candidate Rho-related GTPases, *Dictyostelium* possesses six Rac GTPases (Rac1a, Rac1b, Rac1c, RacB, RacF1 and RacF2), 13 Rac-like GTPases (RacC, RacD, RacE, RacG, RacH, RacI, RacJ, RacL, RacM, RacN, RacO, RacP and RacQ), and one RhoBTB homolog (RacA), but no obvious Rho or Cdc42 family GTPases (Rivero and Xiong, 2016). To identify the Rac isoform(s) that interacts with CARMIL-GAP, its isolated GAP domain (specifically residues 715–858) was expressed as a GST fusion, bound to glutathione Sepharose 4B, and incubated with *Dictyostelium* cell lysates. After extensive washes, the bound material was eluted with high-salt buffer, concentrated, digested with trypsin, and the digest subjected to mass spectrometry/sequence analysis. In terms of Rho-related GTPases, the curated list of bound proteins (Table S1, see the legend for details) contained only two Rho-related GTPases – the Rac-like GTPase RacE (10 distinct peptides) and Rac1 (six distinct peptides; Fig. 2A). With regard to Rac1, although peptides 1, 3 and 4 are present in all three Rac1 isoforms (Rac1a, Rac1b and Rac1c), and peptide 5 is present in both Rac1a and Rac1b, peptides 2 and 6 are present only in Rac1a. These results, together with the fact that Rac1a is expressed at vastly higher levels than Rac1b and Rac1c in both vegetative and starved cells based on RNA measurements (available on dictyExpress; Stajdohar et al., 2017), argue that the GAP domain in CARMIL-GAP interacts preferentially within cells with the 1a isoform of *Dictyostelium* Rac1. Given the phenotype of CARMIL-GAP-null cells (see below), which do not exhibit a defect in cytokinesis when grown in suspension (a process regulated by RacE; reviewed in Rivero and Xiong, 2016), but do exhibit a pronounced defect in phagocytosis (a process regulated by Rac1a; reviewed in Rivero and Xiong, 2016), we focused on the role of the GAP domain of CARMIL-GAP in regulating the nucleotide state of Rac1a.

To obtain direct evidence that the GAP domain in CARMIL-GAP functions to accelerate the rate of GTP hydrolysis by Rac1a, we expressed the following N-terminally tagged GST fusion proteins: full-length Rac1a (GST–Rac1a), the GAP domain (GST–GAP), and a version of the GAP domain in which the conserved arginine residue required for robust GAP activity in known GAPs was changed to an alanine residue (GST–GAP-RΔA). Fig. 2B shows these three fusion proteins following purification. Of note, specific conditions were used to obtain GST–Rac1a in its GTP-bound form prior to performing the GAP assay (see Materials and Methods). To measure the effect of the GAP domain from CARMIL-GAP on the rate of GTP hydrolysis by Rac1a, 10 µM of GST–Rac1a was incubated for 10 min at 20°C with either 2.5 µM GST–GAP or GST–GAP-RΔA, at which point the amount of free phosphate in solution (a measure of GTP hydrolysis by GST–Rac1a) was determined by measuring the absorbance of the dye CytoPhos (see Materials and Methods). Fig. 2C shows that the addition of GST–GAP increased the intrinsic rate of GTP hydrolysis for Rac1a (normalized to a value of 1.0) by an average of 5.8 fold. In contrast, the addition of GST–GAP-RΔA increased rate of GTP hydrolysis for Rac1a by only 1.7 fold. Together, these data argue that the GAP domain in CARML-GAP is functional, and that CARMIL-GAP likely functions as a GAP for Rac1a in *Dictyostelium*.

As discussed in the Introduction, CARMIL proteins are best known for their interaction with and regulation of CP, which is mediated by their CPI domain. Sequence alignment (Fig. 1C) shows that CARMIL-GAP possesses a typical CPI domain containing the invariant arginine residue that is essential for CP interaction (residue 989; highlighted red in Fig. 1C), as well as other residues that potentiate CP binding and regulation (highlighted blue in Fig. 1C) (McConnell et al., 2020; Uruno et al., 2006). To demonstrate that CARMIL-GAP does indeed bind CP, residues spanning its CPI domain (residues 963–1005) were expressed as an N-terminally tagged GST fusion protein (GST-CPI), bound to glutathione Sepharose 4B resin, incubated with *Dictyostelium* cell lysates and, after extensive washes, the bound proteins were eluted with high-salt buffer. As a negative control, a parallel binding reaction was performed using a version of this CPI fusion protein in which the essential arginine residue at position 989 was changed to a glutamate residue (GST–CPI-RΔE). Fig. 2D shows these two fusion proteins following purification. A western blot of the bound proteins probed with an antibody to the α subunit of *Dictyostelium* CP showed that CP was present in the material eluted from GST–CPI (Fig. 2E, lane 1) but not in the material eluted from GST–CPI-RΔE (Fig. 2E, lane 2). These results argue that CARMIL-GAP also functions as a regulator of CP.

### CARMIL-GAP-null cells grow normally in liquid medium and do not exhibit defects in macropinocytosis and cell division

**Fig. 3.**
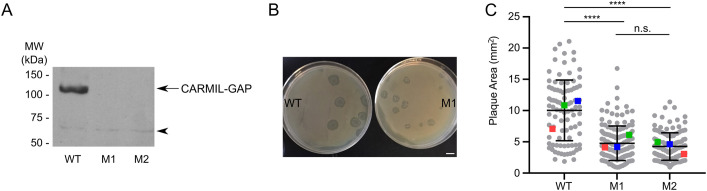
**CARMIL-GAP null cells grown on bacterial lawns make significantly smaller plaques.** (A) Western blot of whole-cell extracts prepared from equal numbers of control AX3 cells (WT) and CARMIL-GAP-null cell lines M1 and M2 probed with an antibody to CARMIL-GAP. The cross reacting band at ∼63 kDa (see arrowhead) serves as a loading control (see also Fig. S3, lanes 1 and 2, which included an actin loading control). Blot shown is representative of three repeats (B) Representative examples of WT and M1 KO cells grown on a lawn of *K. aerogenes* for 5 days (plaque assay). Scale bar: 10 mm. (C) Quantification of plaque size at day 5 for WT, M1 KO and M2 KO cells (see also Table 1). The number of plaques scored over three independent experiments is shown at the bottom of each bar. The error bars (s.d.) and statistics are calculated on the cell-level data points, and the mean values for the three experiments performed are indicated by the red, green and blue squares. ****P<0.0001; n.s., not significant (unpaired two-tailed *t*-test).

Western blots of *Dictyostelium* whole-cell extracts probed with an antibody raised against the C-terminal 19 residues of CARMIL-GAP show that it is expressed in both vegetative cells and starved developing cells (Fig. S2A). This result, together with the biochemical evidence above showing that it likely regulates CP, a central player in actin assembly, and Rac1, a central player in the regulation of actin assembly, argues that CARMIL-GAP might regulate actin-dependent processes occurring in both of these physiological states (e.g. macropinocytosis, cell division and phagocytosis in vegetative cells, and cell motility in starved aggregating cells). To define the physiological significance of CARMIL-GAP in both vegetative and starved cells, and to permit dissection of its *in vivo* functions by complementation, we created CARMIL-GAP-null cells using homologous recombination. Briefly, AX3 cells were transformed with a linear gene disruption fragment containing the selectable marker blasticidin S flanked by portions of the CARMIL-GAP gene, and single, blasticidin S-resistant cells were cloned by serial dilution (see Materials and Methods for details). Fig. 3A, shows a western blot of whole-cell extracts prepared from wild-type (WT) cells and the two independent CARMIL-GAP knockout (KO) cell lines (M1 and M2) that we used interchangeably in this study.

**Table 1.**
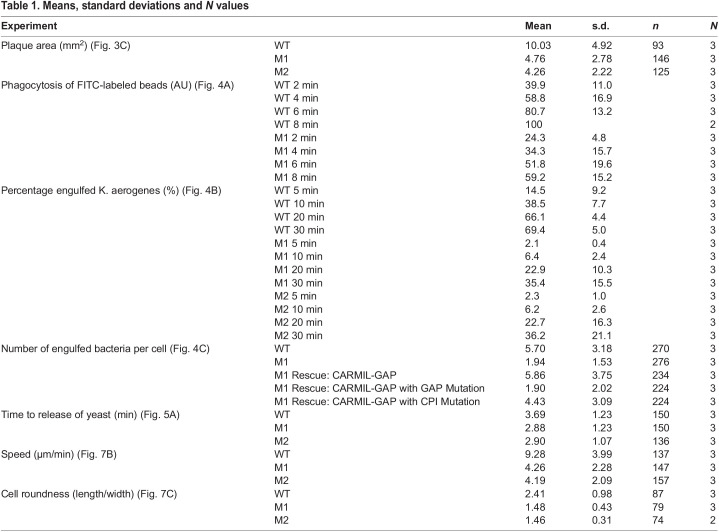
Means, standard deviations and *N* values

In terms of baseline data in vegetative cells, CARMIL-GAP KO cells grew at normal rates in HL5 liquid medium (Fig. S2B). Consistent with this, CARMIL-GAP KO cells exhibited normal rates of macropinocytosis, the mechanism by which axenic strains gain nutrients when grown in liquid medium (Fig. S2C) (Hacker et al., 1997). Moreover, vegetative CARMIL-GAP KO cells did not exhibit a reduction in steady state F-actin content (Fig. S2D). Finally, vegetative CARMIL-GAP KO cells did not exhibit a significant defect in cytokinesis based on measuring the fraction of cells grown in suspension that contain more than one nuclei (Fig. S2E). Taken together, these baseline data indicate that CARMIL-GAP does not play a significant role in either macropinocytosis or cell division, two major actin-dependent processes occurring in vegetative cells.

### CARMIL-GAP-null cells grown on bacterial lawns make significantly smaller plaques, suggesting a defect in phagocytosis

To explore the possibility that CARMIL-GAP plays a significant role in phagocytosis, another major actin-dependent process exhibited by vegetative cells, we initially measured the rate at which cells create bacteria-free plaques when grown in the presence of an even lawn of bacteria as the nutrient source. To accomplish this, WT and CARMIL-GAP KO lines M1 and M2 where seeded at low density with living *Klebsiella aerogenes* bacteria on agar plates made using 5-fold diluted HL5 medium. *Dictyostelium* will not grow on such agar plates alone, so their ability to grow in the presence of the bacteria, which is scored as the size of the plaques that form after 5 days, should be due to their ability to phagocytose the bacteria. Fig. 3B shows that KO line M1 made significantly smaller plaques than WT cells. Consistent with this, quantification showed that both KO lines produced plaques that were ∼35% the size of plaques produced by WT cells (Fig. 3C; see also Table 1). These results suggest that CARMIL-GAP plays a significant role in supporting the actin-dependent process of phagocytosis.

### CARMIL-GAP-null cells exhibit a significant defect in phagocytosis

**Fig. 4.**
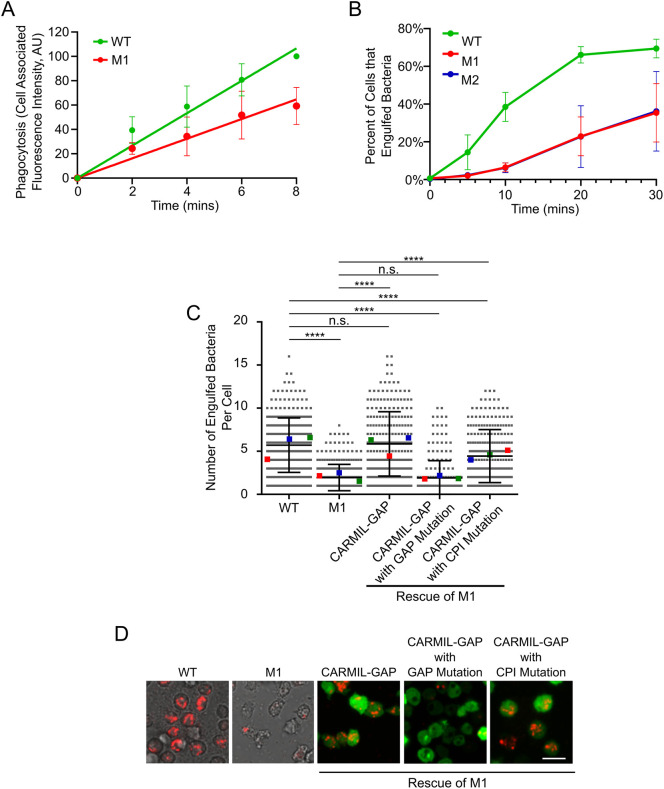
**CARMIL-GAP null cells exhibit a significant defect in the phagocytosis of bacteria.** (A) Shown is the initial rate of uptake by phagocytosis of 1 µm FITC-labeled polystyrene beads by WT and M1 KO cells, presented as cell-associated fluorescence (in arbitrary units; AU) (see also Table 1). (B) Shown are the percentages of WT, M1 KO, and M2 KO cells that had engulfed pHrodo Red dye-labeled *K. aerogenes* by 5, 10, 20 and 30 min, as determined by FACs (see also Table 1). (C) Shown are the numbers of *K. aerogenes* engulfed per cell after 15 min of incubation, as determined by quantitative confocal microscopy, for WT and M1 KO cells, and for M1 KO cells that were complemented with GFP-tagged versions of CARMIL-GAP, CARMIL-GAP containing the GAP domain mutation, or CARMIL-GAP containing the CPI domain mutation (see also Table 1). The error bars (s.d.) and statistics are calculated on the cell-level data points, and the mean values for the three experiments performed are indicated by the red, green and blue squares. ****P<0.0001; n.s., not significant (unpaired two-tailed *t*-test). (D) Shown are representative images of the five cell samples quantified in C (red, bacteria; green, GFP-CARMIL). Scale bar: 10 µm.

The smaller plaque size exhibited by CARMIL-GAP-null cells grown on bacterial lawns could result from defects in processes other than phagocytosis (e.g. a defect in the ability to digest the bacteria; Buckley et al., 2016; Pan et al., 2016). Given this, we sought more direct measures of phagocytic ability. As a first attempt, we measured the initial rate of uptake of fluorescent 1 µm polystyrene beads as the phagocytic substrate. Fig. 4A shows that CARMIL-GAP KO line M1 exhibited a ∼40% decrease in bead uptake over 8 min relative to WT cells (see also Table 1). To provide a more physiological measure of phagocytosis, we used a FACS-based assay (Pan et al., 2018b; Pan et al., 2016) that measures the phagocytosis of pHrodo Red dye-labeled *Klebsiella aerogenes*, whose fluorescence within phagosomes increases dramatically upon the acidification of the phagosome. Fig. 4B shows that the percentage of M1 and M2 cells in suspension that had taken up bacteria over a 30 min incubation was less than half that of WT cells (see also Table 1). Moreover, quantitative confocal imaging (Pan et al., 2018b; Pan et al., 2016) showed that adherent M1 cells internalized ∼67% fewer bacteria than WT cells after a 15 min incubation (Fig. 4C and Table 1; compare ‘M1’ to ‘WT’; see also Fig. 4D for representative images of these two samples). Importantly, expression of CARMIL-GAP (as a GFP fusion) in M1 cells fully rescued this defect in phagocytosis (Fig. 4C and Table 1; compare rescue with ‘CARMIL-GAP’ to ‘WT’; see also Fig. 4D for representative images of these two samples, Fig. S3, lane 3, which shows that these cells make a protein corresponding in size to GFP–CARMIL-GAP, and Fig. S4A1–A4, which shows that GFP–CARMIL-GAP localizes to phagocytic cups in complemented null cells). Together, these results argue that the defect in phagocytosis is indeed caused by the loss of CARMIL-GAP.

**Fig. 5.**
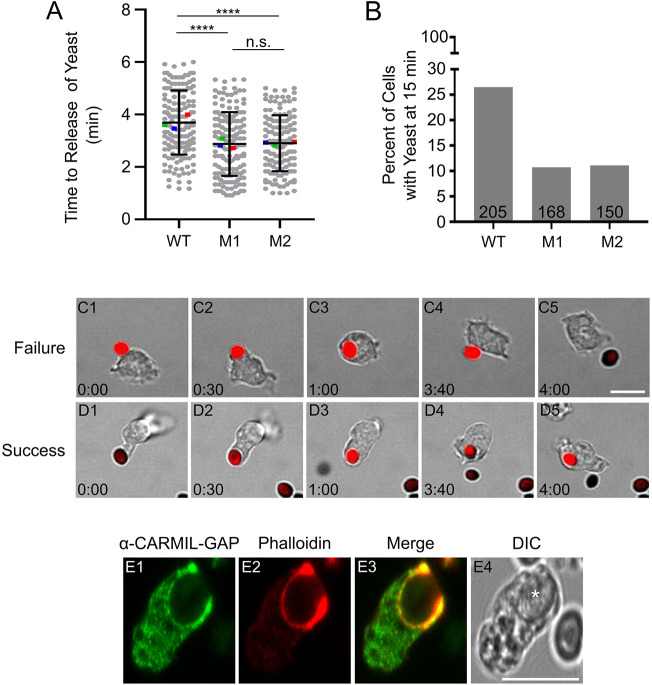
**CARMIL-GAP-null cells exhibit a significant defect in the phagocytosis of yeast.** (A) Shown are times in minutes to release of bound yeast particles for WT, M1 KO and M2 KO cells (see also Table 1). The mean values for the three experiments performed are indicated by the red, green and blue squares. The error bars (s.d.) and statistics are calculated on the cell-level data points. ****P<0.0001; n.s., not significant (unpaired two-tailed *t*-test). (B) Percentage of WT, M1 KO and M2 KO cells that still retained a yeast particle 15 min after initial contact. The total number of cells scored from three independent experiments, is indicated at the bottom of each bar. (C1–C5) Still images taken from a representative movie of a WT cell where a bound yeast particle (red) was released. (D1–D5) Still images taken from a representative movie of an M1 KO cell where a bound yeast particle was successfully internalized. (E1–E4) Image of a representative *Dictyostelium* cell engulfing an unlabeled yeast particle (white asterisk in the DIC image in E4) and stained for endogenous CARMIL-GAP (E1) and F-actin (E2) (E3 shows the merged image). Scale bars: 10 µm.

To supplement these results, we used a confocal microscopy-based assay to follow the phagocytosis of fluorescently labeled yeast, which represent a significantly larger phagocytic substrate than bacteria. Imaging was initiated upon contact with a yeast particle and continued every 5 s for 30 min after contact. For both WT cells and KOs M1 and M2, most of these contacts failed to lead to internalization, with WT, M1 and M2 cells releasing bound yeast particles on average 3.7±1.2, 2.8±1.2 and 2.9±1.1 min (mean±s.d.) after contact, respectively (Fig. 5A; see also Table 1). Fig. 5C1–C5 shows a representative example of such a failed phagocytic event in a WT cell (see also Movie 1). Despite the high failure rate, a subset of WT and KO cells possessed a fluorescent yeast particle 15 min after initial contact, or roughly five times longer than the average time for yeast particle release. Specifically, 26.3% of WT cells (54 out of 205 cells), 10.7% of M1 KO cells (18 out of 168 cells), and 11.3% for M2 KO cells (17 out of 150 cells) still possessed a fluorescent yeast particle 15 min after initial contact (Fig. 5B). Fig. 5D1–D5 shows a representative example of a successful phagocytic event in a M1 KO cell (see also Movie 2). Further evidence that such events correspond to successful phagocytosis events was obtained by examination of every video frame in the final 5 min of the 15 min period for all events judged successful, which showed that the cell-associated yeast particle remained at all times within the 2D footprint of the cell even as it changed shape or migrated (see Materials and Methods for additional details). We conclude, therefore, that the loss of CARMIL-GAP results in a ∼58% decrease in the efficiency of yeast phagocytosis. Finally, staining of WT cells in the process of phagocytosing an unlabeled yeast particle showed that CARMIL-GAP accumulates in the phagocytic cup along with F-actin (Fig. 5E1–E4). Together, the above results indicate that CARMIL-GAP plays a major role in the actin-dependent process of phagocytosis.

### CARMIL-GAP-null cells exhibit a defect in chemotactic streaming

**Fig. 6.**
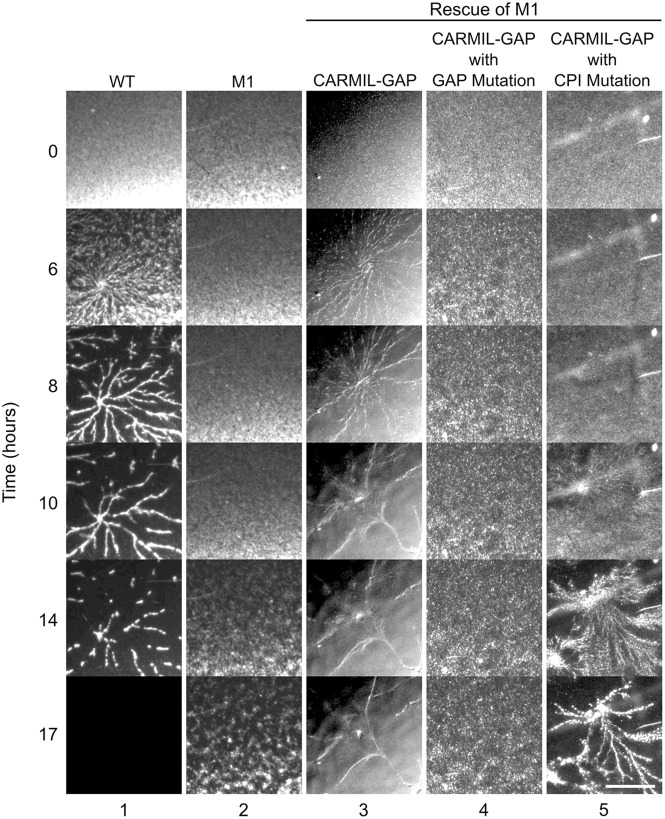
**CARMIL-GAP-null cells exhibit a defect in chemotactic streaming.** Shown are images at 0, 6, 8, 10, 14 and 17 h of streaming assays for WT and M1 KO cells, and for M1 KO cells that were rescued with GFP-tagged versions of CARMIL-GAP, CARMIL-GAP containing the GAP domain mutation, or CARMIL-GAP containing the CPI domain mutation. The images are from a single experiment, and are representative of three independent experiments. Scale bar: 1 mm.

Given that CARMIL-GAP is expressed in starved cells as well as vegetative cells, we next asked whether it plays a significant role in the actin-dependent process of chemotactic aggregation that is initiated by starvation. Specifically, starvation sets in motion a developmental program that drives the coalescence of ∼100,000 cells to form a stalk with a spore-filled head. Cell coalescence is driven by the migration of cells towards an extracellular gradient of cAMP that is generated initially by a small number of pioneer cells. Individual amoeba initially undergo chemotaxis towards these pioneer cells, but within hours begin to merge in head-to-tail fashion to create large streams of cells moving together towards what has now become a cAMP-emitting aggregation center (Ishikawa-Ankerhold and Müller-Taubenberger, 2019; Nichols et al., 2015). Fig. 6, column 1, shows a representative ‘streaming assay’ for WT cells, where large streams had formed by ∼6 h, and aggregation was approaching completion by ∼14 h (see also Movie 3). In sharp contrast, the CARMIL-GAP-null cell line M1 failed to form streams, making only small cell aggregates by ∼17 h (Fig. 6, column 2; see also Movie 4) (a similar result was seen with KO line M2; data not shown). Importantly, expression of GFP–CARMIL-GAP in M1 cells largely rescued this defect in streaming, as large streams were apparent by 8 h (Fig. 6, column 3; see also Movie 5 and Fig. S5A1–A4, which shows that GFP–CARMIL-GAP localizes to the leading edge of complemented, ripple-stage null cells). Of note, a western blot of ‘ripple-stage’ cells (the first observable change for cells seeded at high density on black filters in the absence of nutrients; see below and Katoh-Kurasawa et al., 2021) showed that null cells exhibit approximately normal levels of the cAMP receptor CAR1 (Fig. S6), arguing that their defect in streaming is most likely not due to an inability to sense cAMP (although see Discussion).

**Fig. 7.**
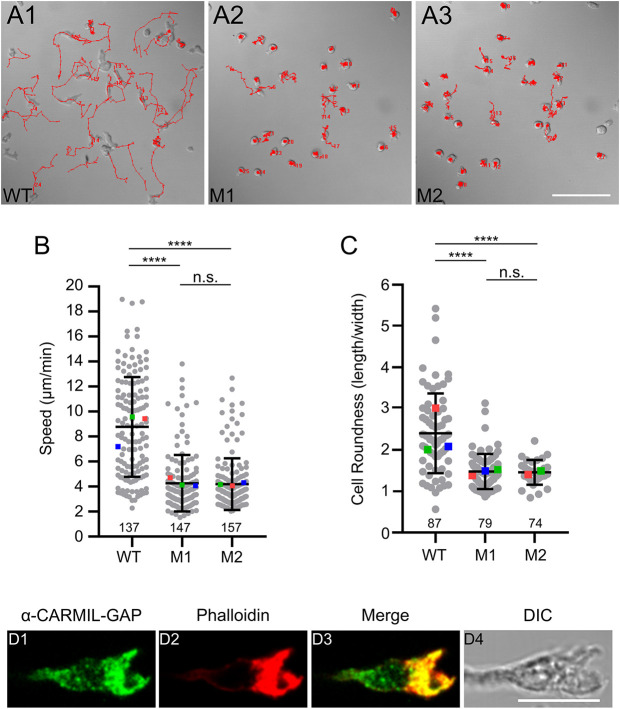
**Ripple-stage CARMIL-GAP-null cells exhibit defects in cell motility and polarization.** (A1–A3) Shown are representative path plots of the random motility exhibited over 15 min by ripple-stage WT (A1), M1 KO (A2), and M2 KO (A3) cells. (B) Speeds of ripple-stage WT, M1 KO, and M2 KO cells (see also Table 1). The number of cells scored from three independent experiments is shown at the bottom of each bar. The error bars (s.d.) and statistics are calculated on the cell-level data points, and the mean values for the three experiments performed are indicated by the red, green and blue squares. (C) Cell roundness values (length/width, with 1.0 being perfectly round) exhibited by ripple-stage WT, M1 KO, and M2 KO cells (see also Table 1). The number of cells scored over three independent experiments is shown at the bottom of each bar. The error bars (s.d.) and statistics are calculated on the cell-level data points, and the mean values for the three experiments performed are indicated by the red, green and blue squares. ****P<0.0001; n.s., not significant (unpaired two-tailed *t*-test). (D1–D4) Image of a representative *Dictyostelium* cell undergoing chemotaxis (to the right) and stained for endogenous CARMIL-GAP (D1) and F-actin (D2) (D3 and D4 show the merged and DIC images, respectively). Scale bars: 100 µm (A3); 10 µm (D4).

To address the underlying cause of the defect in chemotactic aggregation, we starved WT cells and KOs M1 and M2 at high density on black filters until the ‘ripple stage’, which took ∼5 h for both WT and KO cells. At this stage, *Dictyostelium* amoebae exhibit their highest rate of motility, which is about two to four times faster than the rate exhibited by vegetative cells (Hoeller and Kay, 2007; Jung et al., 2016; Jung et al., 1996; Varnum et al., 1986). Ripple-stage cells were harvested by trituration, allowed to attach at low density on chamber slides, and the centroid of every cell in the field of view determined every 15 s for 15 min to obtain motility rates. Representative path plots for WT cells and CARMIL-GAP-null cell lines M1 and M2 (Fig. 7A1–A3, respectively) suggest that CARMIL-GAP-null cells are significantly slower (see also Movies 6, 7 and 8). Indeed, quantification showed that the average rate of motility for CARMIL-GAP null cells was 45.2% (M1) and 44.1% (M2) that of WT cells (Fig. 7B; see also Table 1).

During chemotactic aggregation, the fast speed of amoeba is associated with an elongated, highly-polarized shape that aligns with the direction of migration. Higher magnification images of individual cells in the streaming assays performed in Fig. 5 showed that CARMIL-GAP-null cells appeared on average to be much less polarized than control cells. To quantify this, we measured the ratio of cell length to cell width using still images from the movies used to determine the motility rates of ripple stage cells. Fig. 7C shows that both KO M1 and KO M2 were indeed significantly less polarized than WT cells (see also Table 1). Finally, we found that endogenous CARMIL-GAP concentrated along with F-actin in the leading edge pseudopods of chemotaxing cells, as expected (Fig. 7D1–D4).

### Although the CPI and GAP domains of CARMIL-GAP both contribute to its function, the GAP domain plays a more significant role

To define the relative contributions that the CPI domain (CP regulation) and GAP domain (Rac1a regulation) of CARMIL-GAP make to its overall function, we complemented CARMIL-GAP-null cell line M1 with two mutant versions of CARMIL-GAP expressed as GFP fusions. In one version referred to as ‘CARMIL GAP with GAP mutation’, the invariant arginine residue that is required for robust GAP activity was changed to a function-blocking alanine residue. In the other version referred to as ‘CARMIL-GAP with CPI mutation’, the invariant arginine residue that is essential for CP binding was changed to a function-blocking glutamate residue. With regard to the phagocytosis of bacteria, Fig. 4C shows that the GAP domain mutant was completely incapable of rescuing the CARMIL-GAP-null cell line M1, while the CPI domain mutant partially rescued these cells (the value obtained was in between, and significantly different from, the values for both WT cells and null cell line M1; see also Table 1). With regard to chemotactic streaming, column 4 in Fig. 6 shows that the GAP domain mutant appeared completely incapable of rescuing the CARMIL-GAP-null cell line M1 (see also Movie 9), whereas column 5 in Fig. 6 shows that the CPI domain mutant partially rescued these cells [they did make large streams but took much longer (∼14 h) to do so; see also Movie 10]. Importantly, both mutant GFP–CARMIL-GAP proteins localized to phagocytic cups (Fig. S4B1–B4,C1–C4) and the leading edge of crawling ripple-stage cells (Fig. S5B1–B4,C1–C4). Moreover, both mutant proteins were expressed in complemented null cells at levels exceeding that of WT GFP–CARMIL-GAP in complemented null cells, which exhibit complete rescue (Fig. S3; compare the GFP–CARMIL-GAP signal in lanes 4 and 5 to lane 3). These results argue that the inability of the GAP mutant to rescue either phagocytosis or streaming, and the inability of the CPI mutant to fully rescue these behaviors, is due to their functional defects rather than to mislocalization or insufficient expression. We conclude, therefore, that although both domains contribute significantly to CARMIL-GAP function, the GAP domain plays the more important role. This conclusion adds to the growing evidence that CARMIL proteins regulate actin dynamics by regulating signaling pathways as well as CP, and that the continued cycling of Rho GTPases between their GTP and GDP bound states through the coordinated action of their GEFs and GAPs and is often required to drive Rho-dependent biological processes forward.

## DICUSSION

CARMIL proteins serve as important regulators of actin-dependent cellular processes by virtue of their ability to regulate CP (Edwards et al., 2014; Mullins et al., 2018). Evidence is accumulating, however, that they also regulate these processes by interacting with signaling molecules (Stark et al., 2017). Here, we showed that *Dictyostelium* CARMIL-GAP is responsible for such dual regulation but as a single protein. Moreover, we determined the functional significance of these two activities by complementing CARMIL-GAP-null cells with versions of CARMIL-GAP that lack either GAP activity toward Rac1a or the ability to regulate CP. Although both activities were found to contribute significantly to the ability of CARMIL-GAP to support phagocytosis and chemotactic streaming, its GAP activity towards Rac1a was the more important of the two. Specifically, CARMIL-GAP lacking GAP activity was completely incapable of rescuing the defect in phagocytosis and yielded only a very modest rescue of the defect in chemotactic streaming, whereas CARMIL-GAP lacking the ability to regulate CP rescued both processes to a significant extent, although not completely. For this particular CARMIL, therefore, its ability to regulate a signaling pathway contributes more to its overall cellular function than its ability to regulate CP. Although this conclusion highlights the functional significance of CARMIL-dependent effects on signaling pathways, the relatively modest role played by the CPI domain of CARMIL-GAP should be considered in the context of possible functional redundancy, as CARMIL-GAP-null cells still contain CARMIL and at least one other protein containing a CPI domain (see the legend to Fig. S1 for details). It is also important to note that although the discussion below focuses on the role of the GAP domain of CARMIL-GAP in regulating Rac1a, we cannot exclude the possibility that this domain regulates additional Rho-related GTPases (e.g. RacE), and that their misregulation contributes to the defects in actin-dependent processes exhibited by CARMIL-GAP-null cells. In a similar vein, we cannot exclude the possibility that the profound defect in streaming exhibited by cells lacking the GAP activity of CARMIL-GAP is due at least in part to the misregulation of GTPases required for progression of the developmental program in *Dictyostelium*. The pronounced defect in phagocytosis exhibited by null cells cannot be attributed, however, to defects in this developmental program.

Our study focused on Rac1a as the target of the GAP activity of CARMIL-GAP, as two of the six Rac1-related peptides we obtained were specific to this isoform, and none were specific to Rab1b or Rac1c . That said, Rac1b and Rac1c could also be targets given that four of the six peptides in our mass spectrometry data are also present in these two isoforms. Any GAP activity towards Rac1b and Rac1c would probably not be that consequential, however, as Rac1a is expressed at vastly higher levels than Rac1b and Rac1c in both vegetative and starved cells based on RNA measurements (available on dictyExpress; Stajdohar et al., 2017).

We assume that the GAP domain of CARMIL-GAP functions together with one or more GEF partner(s) to regulate the nucleotide state of Rac1a in such a way as to promote phagocytosis and chemotactic streaming (*Dictyostelium* contains 46 genes encoding conventional RhoGEFs; Rivero and Xiong, 2016). With regard to the protein or proteins downstream of Rac1a whose activity is regulated by the GAP activity of CARMIL-GAP, one major candidate is the pentameric WAVE regulatory complex (WRC), which triggers the formation of branched actin networks by the Arp2/3 complex by coupling active Rac in the plasma membrane to the activation of SCAR/WAVE proteins, which acts as nucleation-promoting factors (NPFs) for the Arp2/3 complex (Davidson and Insall, 2011; Davidson and Insall, 2013; Dumontier et al., 2000; Filic et al., 2012; Mullins et al., 2018; Pollitt and Insall, 2009; Rivero and Xiong, 2016; Rotty et al., 2013; Schaks et al., 2019; Swaney and Li, 2016). This pathway likely underlies the effect that the GAP activity of CARMIL-GAP has on the process of chemotactic streaming, as the leading-edge pseudopods driving streaming in *Dictyostelium* are known to be created by Arp2/3 complex-dependent branched actin nucleation downstream of Rac1, the WRC and the SCAR/WAVE NPFs (Davidson et al., 2018; Rivero and Xiong, 2016; Schaks et al., 2018; Veltman et al., 2012). This pathway might also underlie the effect that the GAP activity of CARMIL-GAP has on the process of phagocytosis, since Rac1 also contributes to the formation of the branched actin networks that comprise much of the phagocytic cup [in this case through the NPF WASP (Davidson et al., 2018; Gotthardt et al., 2006; Jeon and Jeon, 2020) as well as the SCAR/WAVE NPFs (Seastone et al., 2001)]. Finally, the GAP activity of CARMIL-GAP might contribute to phagocytosis and chemotactic streaming by regulating additional downstream effectors of Rac1 (e.g. PAK kinases, formins or IQGAP; Buckley et al., 2016; de la Roche et al., 2005; Rivero and Xiong, 2016).

An obvious question is why the deletion of CARMIL-GAP does not actually promote phagocytosis and chemotactic streaming given that GAPs push Rho-related GTPases into their GDP-bound ‘off’ state. Indeed, past studies have raised similar questions regarding the regulation of Rho-dependent cellular processes by GEF-GAP pairs based on the simplified view that the GEFs should promote the process by increasing the amount GTP-bound Rho, while the GAPs should inhibit the process by decreasing the amount of GTP-bound Rho. An early ‘crack’ in this simplified view came from studies showing that dominant-active versions of Rho-related GTPases often cannot support biological processes (reviewed in Parrini and Camonis, 2011). This crack has continued to widen, as numerous mechanistic studies have revealed a variety of ways in which GEF and GAP activities are both employed to promote Rho-dependent cellular processes (reviewed in Denk-Lobnig and Martin, 2019). For example, instances exist where both activities act at the same time in different places, at different times in the same place, and at the same time and in the same place. Importantly, these variations yield variations in the temporal and/or spatial control of the nucleotide state of the Rho GTPase that are required by specific biological processes. For example, oscillations in the nucleotide state of RhoA created by the sequential actions of a RhoA GEF and a RhoA GAP drive the pulsatile contractions of medioapical actomyosin networks that are required for the apical constriction of epithelial cells (Mason et al., 2016). The one constant in all of these studies is that GEFs and GAPs are both required in some fashion or another for the proper execution of Rho-dependent biological processes.

One example of GEF-GAP coordination that is particularly relevant to our study was provided by Schlam and colleagues (Schlam et al., 2015), who showed that the phagocytosis of large IgG-coated particles by macrophages requires the activity of several GAPs for Rac1 and Cdc42, whose activation by GEFs is also required for Fc receptor-mediated phagocytosis. In this case, the GAPs appeared to be driving actin network disassembly at the base of the phagocytic cup even as the cup was still undergoing GEF-dependent pseudopod extension around the particle. The authors suggested that this localized GAP-dependent actin network disassembly serves to promote phagocytosis by recycling limiting components to the growing tips of advancing pseudopods, as well as by clearing a path for final particle internalization. In many ways, then, this is an example of ‘same time, different place’ GEF-GAP coordination. The defect in the ability of CARMIL-GAP-null cells to phagocytose a large particle (yeast), where events commonly reversed part way through the engulfment process, seems in line with the results of Schlam et al., although CARMIL-GAP is not restricted to the base of the phagocytic cup like the GAPs imaged in that study.

Another common form of GEF-GAP coordination is having them act at the same time and in the same place. Although this might seem at first sight to create a futile cycle, what it actually creates is a dynamic cycling of the nucleotide state of the Rho protein between its active and inactive forms (Denk-Lobnig and Martin, 2019). Importantly, such dynamic cycling can be employed by the cell to tune Rho signaling to an optimal level, as well as to rapidly adjust this level to meet varying functional demands. The RhoA-dependent regulation of the actomyosin flows that establish left–right asymmetry in *C. elegans* embryo (Schonegg et al., 2007; Schonegg and Hyman, 2006) and the Rac1-dependent regulation of the branched actin networks that define dendritic spine morphology (Um et al., 2014) represent examples where Rho-related GTPases are controlled by GEFs and GAPs that colocalize and function simultaneously. By analogy, CARMIL-GAP and its partner GEF might function at the same time and in the same place to control the Rac1a-dependent assembly and subsequent turnover of the branched actin networks that comprise the phagocytic cup and the leading edge of migrating cells.

Assuming CARMIL-GAP and its partner GEF do function at the same time and in the same place to drive the rapid cycling of nucleotide state of Rac1a, how might this serve to promote particle engulfment and cell migration? In thinking about this question, it is important to consider the accumulating evidence that CARMIL proteins shuttle between two states – a folded, inactive form in the cytoplasm that cannot regulate CP, and an unfolded, active form at the plasma membrane that can regulate CP (Fujiwara et al., 2014; Uruno et al., 2006). Although the molecules that recruit CARMIL proteins to the plasma membrane have not been identified with absolute certainty, one likely candidate for vertebrate CARMILs is active Rac1, which would interact with these CARMILs via their CRIB domain-like sequences (Fujiwara et al., 2014). Importantly, the recruitment of CARMILs to the plasma membrane by active Rac (and possibly other molecules like polyphosphoinositides) is thought to trigger their unfolding and activation, thereby allowing them to begin converting sequestered CP (CP bound to V-1) into CARMIL – CP complexes that then cap nascent branched actin filaments (Fujiwara et al., 2014; Mullins et al., 2018). If CARMIL-GAP were also recruited to the plasma membrane by active Rac1a, then its GAP activity towards Rac1a could drive repeated rounds of CARMIL-GAP recruitment to, and release from, the plasma membrane when combined with the simultaneous activity of a Rac1a GEF. Given the likelihood that membrane-bound CARMIL-GAP also collaborates with V-1 in *Dictyostelium* (Jung et al., 2016) to drive the capping of nascent filament barbed ends required for branched actin network formation, then the rapid cycling of CARMIL-GAP on and off the plasma membrane might serve to promote the advance of pseudopods during phagocytic particle engulfment and leading edge extension.

## MATERIALS AND METHODS

### Cell biological methods

*Dictyostelium* strain AX3 was grown in HL5 medium, transformed by electroporation, and stable clonal transformants were isolated as described previously (Jung et al., 1996). The growth of *Dictyostelium* on plates containing *Klebsiella aerogens* bacteria (plaque assay) was performed as described previously (Jung et al., 1996). The initial rate of uptake of 1 µm FITC–latex beads by phagocytosis, and the rate of macropinocytosis of FITC–dextran, were performed as described previously (Jung et al., 2001). Streaming assays, measurements of the speed and polarity of ripple-stage cells, measurements of growth rates and the number of nuclei per cell, and measurements of cellular F-actin content by FACs analysis of cells stained with FITC-phalloidin, were all performed as described previously (Jung et al., 1996). The preparation of whole cell extracts, SDS-PAGE, and western blotting were performed as described previously (Jung et al., 2001). Fixing, immunostaining and imaging *Dictyostelium* on a Zeiss LSM 780 microscope equipped with a 63×1.4-NA objective (or a 40×1.2 NA for the motility assays) were performed as described previously (Jung et al., 1996).

### Vectors

WT CARMIL-GAP, CARMIL-GAP containing the arginine to alanine point mutation at residue 737 in the GAP domain, and CARMIL-GAP containing the arginine to glutamate point mutation at residue 989 in the CPI domain, were cloned with EcoR1 ends using standard techniques, sequenced confirmed, and expressed as GFP fusions using the vector pDEX H (Jung et al., 1996) Transformation and the selection of stable transformants using G418 were performed as described previously (Jung et al., 2016).

### Reagents

The polyclonal antibody to the αsubunit of *Dictyostelium* CP was a generous gift of John A. Cooper (Washington University, St Louis, USA). Blasticidin-S and G418 were purchased from Sigma. Labeled phalloidins and secondary antibodies and the protein molecular weight marker were purchased from Thermo Fisher Scientific. The mouse monoclonal antibody against CAR1 was a generous gift of Carol Parent (National Cancer Institute, Bethesda) and was used at 1:100. Protein concentrations were determined by Bradford assay (Bio-Rad).

### CARMIL-GAP antibody

A peptide corresponding to CARMIL-GAP residues 939–953 was synthesized, conjugated with keyhole limpet hemocyanin (KLH), and injected into rabbits using standard techniques (Wang et al., 2010). Rabbit sera obtained after primary immunization and three boosts was purified by absorption against CARMIL-GAP-null cell extracts as previously described (Jung et al., 1996).

### CARMIL-GAP knockout

A linear gene-disruption fragment designed to promote a double-crossover gene replacement event was created by fusing nucleotides 1080–1591 and nucleotides 2193–2715 of the *Dictyostelium* CARMIL-GAP genomic sequence (DictyBase Gene ID No. DDG_G0290439) to the 5′ and 3′ ends of the Blasticidin resistance cassette in plasmid Bsr2 (Sutoh, 1993), respectively, using the same approach as described previously for knocking out *Dictyostelium* MyoJ (Jung et al., 2009). The introduction of this linear fragment into AX3 cells by electroporation, and the isolation of Blasticidin-resistant clones by serial dilution in 96-well plates, were performed as described previously (Jung et al., 2009) CARMIL-GAP-null cell lines M1 and M2 were identified by western blotting using the CARMIL-GAP antibody.

### GAP domain pulldown

The GAP domain of CARMIL-GAP (residues 715–858) was synthesized by Blue Heron Inc. as an EcoR1/Xho1 fragment using *E. coli* codon bias and a QSGAG spacer between GST and the protein, and then cloned into pGST-Tev Parallel #2 using standard techniques to create GST–GAP. The expression of the GST–GAP fusion protein in *E. coli* strain BL-21-RILP (Stratagene) and its purification using glutathione Sepharose 4B were performed as described previously (Jung et al., 2001; Jung et al., 2016). For the pulldown, glutathione Sepharose 4B beads loaded with GST–GAP were incubated at 4°C for 2 h with a *Dictyostelium* whole-cell extract that had been dialyzed into 1× Tris-buffered saline (TBS) containing 5 µM GTPγS. After five washes with 1× TBS, bound proteins were eluted with high-salt buffer (5× TBS), concentrated using a Ultracel-10 (10 kDa) Amicon filter, and subjected to mass spectrometry analysis.

### Liquid chromatography tandem mass spectrometry analysis

Protein identification by liquid chromatography tandem mass spectrometry analysis (LC-MS/MS) analysis of peptides was performed using an Orbitrap Fusion Lumos Tribid mass spectrometer (Thermo Fisher Scientific, San Jose, CA, USA) interfaced with an Ultimate 3000 Nano-HPLC apparatus (Thermo Fisher Scientific). Peptides were fractionated by EASY-Spray PepMAP RPLC C18 column (2 μm, 100A, 75 μm×50 cm) using a 120-min linear gradient of 5–35% acetonitrile in 0.1% formic acid at a flow rate of 300 nl/min. The instrument was operated in data-dependent acquisition mode using Fourier transform mass analyzer for one survey MS scan on selecting precursor ions followed by 3 s data-dependent higher-energy collisional dissociation (HCD)-MS/MS scans for precursor peptides with 2–7 charged ions above a threshold ion count of 10,000 with normalized collision energy of 37%. Survey scans of peptide precursors from 300 to 2000 *m*/*z* were performed at 120k resolution and MS/MS scans were acquired at 50,000 resolution with a mass range *m*/*z* of 100–2000.

### Protein identification and data analysis

All MS and MS/MS raw spectra from each set were processed and searched using Mascot algorithm within the Proteome Discoverer 1.4 (PD 1.4 software, Thermo Fisher Scientific). Precursor mass tolerance was set at 20 parts per million (ppm) and fragment ion mass tolerance was set at 0.05 Da. Trypsin was selected as the enzyme, with two missed cleavages allowed. Carbamidomethylation of cysteine was used as fixed modification. Deamidation of glutamine, deamidation of asparagine and oxidation of methionine were used as variable modifications. The *Dictyostelium discoideum* sequence database from SwissProt was used for the database search. Identified peptides were filtered for a maximum 1% false discovery rate (FDR) using the Percolator algorithm in PD 1.4 along with additional peptide confidence set to medium. The final lists of protein identification/quantitation were filtered by PD 1.4 with at least three unique peptides per protein identified with medium confidence. For the quantification, a label-free approach was used, where the area under the curve for the precursor ions is used to calculate the relative fold change between different peptide ions.

### CPI domain pulldown

The CPI domain of CARMIL-GAP (residues 965–1005) was synthesized by Blue Heron Inc. as an EcoR1/Xho1 fragment using *E. coli* codon bias and a QSGAG spacer between GST and the protein, and then cloned into pGST-Tev Parallel #2 using standard techniques to create GST–CPI. A second version in which the essential arginine at position 989 was changed to a glutamate residue (GST–CPI-RΔE) was also synthesized. The expression of the GST–CPI and GST–CPI-RΔE fusion proteins in *E. coli* strain BL-21-RILP and their purification using glutathione Sepharose 4B were performed as described previously (Jung et al., 2001). The pulldown of CP using these two fusion proteins was performed exactly as described previously for the pulldown of CP by GST-V1 (Jung et al., 2016).

### GAP assay

*Dictyostelium* Rac1a was synthesized by Blue Heron Inc. as an EcoR1/Xho1 fragment using *E. coli* codon bias and a QSGAG spacer between GST and the protein, and then cloned into pGST-Tev Parallel #2 using standard techniques to create GST–Rac1a. The expression of the GST-Rac1a in *E. coli* strain BL-21-RILP its purification using glutathione Sepharose 4B were performed as described previously (Jung et al., 2016; Jung et al., 2001). GAP assays were performed as described previously (Faix et al., 1998) except that GTP hydrolysis was quantified using CytoPhos reagent (Cytoskeleton Inc.) to measure the amount of free phosphate. Briefly, purified GST–Rac1a was converted into its GTP-bound form by incubation with a 50-fold molar excess of GTP in the presence of 40 mM EDTA and 200 mM (NH4)_2_SO4 at 4°C for 1 h, desalted into 20 mM Tris-HCl (pH 7.5), 5 mM MgCl_2_ and 1 mM DTT to remove free nucleotide, and concentrated by Amicon filtration. The amount of phosphate released by GTP hydrolysis after a 10 min incubation at 20°C was determined for Rac1a alone, Rac1a plus GST–GAP and Rac1a plus GST–GAP-RΔA using CytoPhos reagent according to the manufacturer's instructions.

### Bacteria phagocytosis assays

The FACS-based phagocytosis assay employing pHRodo Red-labeled *Klebsiella aerogenes* (which fluoresce brightly when subjected to the low pH inside acidified phagolysosomes) was performed exactly as described previously (Pan et al., 2018 a; Pan et al., 2016). Briefly, *Klebsiella aerogenes* labeled with pHrodo Red dye (Life Technologies) were incubated at 22°C and 150 rpm with WT and CARMIL-GAP KO cells suspended in phosphate buffer (7.4 mM NaH_2_PO4⋅H_2_O, 4 mM Na_2_HPO_4_⋅7H_2_O, 2 mM MgCl_2_, 0.2 mM CaCl_2_, pH 6.5) at a ratio of ∼100 bacteria per *Dicytostelium* cell. At the indicated times, *Dictyostelium* cells were pelleted by centrifugation, resuspended in an alkaline buffer (50 mM Tris-HCl pH 8.8, 150 mM NaCl) to quench any possible fluorescence coming from non-engulfed bacteria, and the fluorescence signal for pHrodo Red inside *Dictyostelium* was determined by flow cytometry using a FACSort flow cytometer (BD Bioscience), Cell Quest software (v. 3.3) and the FlowJo analysis program (v. 10.0.8; Tree Star). The imaging-based phagocytosis assay employing pHRodo Red-labeled *Klebsiella aerogenes* was also performed exactly as described previously (Pan et al., 2016; Pan et al., 2018a). Briefly, *Dictyostelium* cells were allowed to attach to chamber slides (Lab-Tek) and then incubated with pHrodo Red-labeled bacteria in phosphate buffer at a ratio of about one *Dictyostelium* to 50 bacteria. After 15 min, the bacteria-containing buffer in the chamber slide was replaced with the alkaline buffer described above to halt further bacteria engulfment and quench any fluorescence coming from un-engulfed bacteria. The number of engulfed bacteria per *Dictyostelium* cell was then determined by imaging using a Zeiss LSM 880 confocal microscope equipped with 60×1.3 NA Plan-Neofluar objective lens.

### Yeast particle phagocytosis

Heat-killed *S. cerevisiae* (Invivogen) were labeled with TRITC as described previously (Rivero and Xiong, 2016) *Dictyostelium* cells were mixed 1:5 with TRICT-labeled yeast particles in HL5 medium and placed in a chambered cover glass. After 10 min at 20°C, the capture of differential interference contrast (DIC) and fluorescence images was commenced at 5 s intervals for 25 to 35 min using a Zeiss LSM 780 equipped with a 40×1.2 NA objective. For each event scored, time zero corresponded to contact between a *Dictyostelium* cell and a yeast particle. Failed phagocytic events, which represented the bulk of events scored, corresponded to the subsequent separation of the yeast particle from the *Dictyolstelium* cell. Successful phagocytic events were defined by two criteria: (1) the *Dictyolstelium* cell and the yeast particle remained together for at least 15 min (or roughly five times longer than the average time to failure), and (2) examination of every video frame in the last 5 min of the 15 min period for every event judged successful showed that the cell-associated yeast particle remained at all times within the 2D footprint of the *Dictyostelium* cell even as it changed shape or migrated.

### Statistics

Statistical significance was determined using unpaired two-tailed *t*-test and indicated as follows: *P<0.05, **P<0.01, ***P<0.001 and ****P<0.0001. The values for ‘*n*’ (the number of cells or samples scored) and ‘*N*’ (the number of independent experiments performed) are provided in Table 1 or the figure legends.

## Supplementary Material

Supplementary information

Reviewer comments
