# Supplementary material for: Dual regulation of the actin cytoskeleton by CARMIL-GAP
Source: J Cell Sci. 2022 Jun 20;135(12):jcs258704. doi: 10.1242/jcs.258704 (PMC9270954; doi:10.1242/jcs.258704)
Supplement: Supplementary information [file joces-135-258704-s1.pdf]

```
CARMIL          --MSEEISPNDRKFITNLLTQKNQESLLLSIGDKISKKKNKKPSKRIILITKNRIFFLKP 58
                     .:|  ::| | .:||:|::|. |::    : ::|:|| . : ::::.||||::.
CARMIL-GAP      MTTPVSLSQVEKKTIESILTNKKEEGKLITKVTIL-NEKKKKLEIKTLILSSNRIFLFTD 59

CARMIL          SQNKVKKDIHLLDIQEIKSSTSNEFTIVAKVDNKQFSYGLITNKTDEIINQIRVTFNHQF 118
                |:.|:. : | |:| || |.:.|:.||  | :|      .    .:.|::: :  |:   |
CARMIL-GAP      SKGKLSAEHHYLEITEIASQSENDVTIKFKNQNVMK-MN---LDASEVLKTLYGTLMSTF 115

CARMIL          FGCPEESTFKCTDIKDSRLV-----EIEQKDLPCGGFVETYQSICDHLGVPPRDDICWDM 173
                 |    .|:  .    ||:         :.   ||.|  ||:|:|||||| | ..| ||:
CARMIL-GAP      PGIKIGKTIIFNITPASRIPSIFQATSFKDIQGCGSFNLTYRSVCDHLGVQPLSSILWDI 175

CARMIL          TNIISSKNIRSFNIGEIELPTSAGDTIRCLLGALKYNNYFKSFNFNNYTFNKEQFGYLAE 233
                       ::|.||:.||     :   ::.:| :|.||.||:||: |.  :.:|::. |||
CARMIL-GAP      ENLYPFNQVREFNLAEIYQ--YSFTDLKAILLSLSYNTYFQSFQSNGK-LSNEDLNLLAE 233

CARMIL          VLKCNSTVEDLSLNNVGLKHDTMPIIATALSSNKNLALTAIDISNNQIEDKGMTAFSSYV 293
                |:| ||:::::|::.|   .:: :  :   ::.|| | |: |::.||.:|:||: ::.| :
CARMIL-GAP      VFKENSSLQQLTITNSQASKEPIITLLQNVAENKALHLSHINLNNNSLENKGILTLGSSI 293

CARMIL          ASSLRGIASLDVSNTNCNKAGISVLTNALKKNIKMSSTLSYLNLSGNKMEADGSAGLSSF 353
                 :  .|:: |::.||.|.  |:..: ..|. |  :..:::||:|.||:|: |: ||..|
CARMIL-GAP      QTFSNGLTYLNIENTSCSGKGLEAMFGCLTANNVVCGSMTHLNISNNKLESYGTNGLCKF 353

CARMIL          LASPNTLKTLNISNTTPSMETIVGALVIGCAELKTIDISDNKLT--KKEVPHLVRFIGAS 411
                |:. .:| : | :|||:|  . :     ..:.::|:|:|.|| |  |:.| .:: |:
CARMIL-GAP      LSKATALQQLLMSNTSPVYSFLK----TSSSSIQTLDFSGNKSTTTKESVIDILSFFKQM 409

CARMIL          STLKHFNLSGTKVPVENLKELVVAITSNIYLQDVVLDLKNNDLGIAGARMLASLATDKLS 471
                | |: .| ||  :.  ::|||                             |  : .|
CARMIL-GAP      SHLQTVNLSRIQTTPDDLKLL-----------------------------FS-PATSLL 438

CARMIL          NVIYLDVSENDFGDEGVSVICDGFVGNSTIKKLILNGNFKQSKTKSRPSAIESVISLLES 531
                :   :|:||||:|| |:  :|: :  ||.:::| ::||||  |:| |  |||::|.|::.
CARMIL-GAP      KCSSVDLSENDLGDGGIIKLCEIMYPNSNLHHLSIDGNFKT-KSKLRARAIEALINLIDD 497

CARMIL          ECPLETLHMTVGNSKSPLKADILSLIYSLATNSSLLELDISGHQMGPKGAIGLGKALQTN 591
                :  :|:| :: |..|  ||.|:: |: || .|.||::|||||: :| .||:.:.| | .|
CARMIL-GAP      NTSIESLSLASGSGKYQLKGDLMPLLLSLLKNQSLVKLDISGNGVGDSGALAVSKILWKN 557

CARMIL          KTLHTLIWDDNLTTAIGFAGFQVGLERNL-TLKNMPTPLNDIIQCHREPKFQQ----IWK 646
                :||::|  |.|  :  .:  :: .:.||  :|. :  ||.||    :: .  .
CARMIL-GAP      QTLKSLKCDGNDFSYTALKMIKCSIRRNEKSLTILQLPLSDISTILKNDSNGSSHEK--- 614

CARMIL          EIDSCINRNQSPTRAFEGNGGNSIGATNLSFLASGQQQGVEKLLNKIKSIGRK--VTDPN 704
                                                          :|:.|:.|  .  :. ||
CARMIL-GAP      -----------------------------------------ILKTIQDIQTSIVINYPN 632

CARMIL          NILIVKDAESTEKVIGGIHLIKESIHASLEMELNQKLKDFVQVVNDVINAKKNEMTQQIL 764
                ||  :      :.  ||:
CARMIL-GAP      NIHPI------DTSSGGLT----------------------------------------- 645

CARMIL          ESMQNTFQSMDGPTIKRLATTIQY--------------GSKDVDEQQIHSTLVKG---- 805
                           . |:|  || .|:                | : : ::  :. |  |
CARMIL-GAP      --------PTKQPSIGSLAGVIKKPPIGPPSTPTKSSNPGFQTLPKRPTRGPLYPGRATG 697

CARMIL          AGAELSSRAHECFISALDIASDYTYEKITIGLDSVFKDLILEESQAQNEASGATP----- 860
                ... |:.:|    |.                || |
CARMIL-GAP      GAVTLTDKA---QIN-----------ASTIELP---------------------PLVSR 721

CARMIL          ------------------------------------------------------------
                
CARMIL-GAP      SIDLLMEQGISSVGIFRTCASATALKKIKTRFEAGEDIDLKAENVDVDTVAGVLKSYFRE 781

CARMIL          ------------------------------------------------------------
                
CARMIL-GAP      LPNPIFPENLHEYFFQAMRQSSNEEIIQSLKDIIDQLSPLECKMIKKLFHLLHLISLEKD 841

CARMIL          -------------------------------------IPD---SPVPTR--------S- 870
                                                       | |    .|: :          |
CARMIL-GAP      VNMMSPENIAICWAPTFFRSFASELLPINSFMIVNYFDIFDPENKPISSDNSGDADTDST 901

CARMIL          -----------PQPTSPPITPQPTPTTNVPPVTAPRTGAAAPLKPANPPPVSTTTTPPV 918
                               | ||:|. |||..:  |     .                    |
CARMIL-GAP      QSTCTTNNNNLVATSLSSPISPSTTPTKSIDNVLHTTV-------------------EV 941

CARMIL          STTPKPTQPVSKFGAKLSANSAVAEAIARNMGGGAPPIRKPVAPEPEPEPVTPTKDVTPL 978
                | :|                |        |                        :: | |:
CARMIL-GAP      SRSP--------------NHE------R------------------------AHTVGPM 955
CARMIL          KSKPVVAPRSTPTTSTPTKTPVKKPSGPSVP------GSLSDAPESDSAELTHVT-ASRP 1030
                 ||                      .|              | :. |  :..   |.|  |||
CARMIL-GAP      TSK--------------------RP------ISNNNGVSSNTPPLPNNVTPHHNTMPSRP 990

CARMIL          HIASKRKPPTRRPRPPTEN----------------------------------------- 1050
                  :  ::||:
CARMIL-GAP      SASPIKRPPSMGGSLSNFAGIPLSNHSSSGQLNNNNSNNNTTSNSSNSSSGKSSSNPSPI 1050

CARMIL          ------------------------------------------------------------

CARMIL-GAP      PTPPDSPSMSYIGDGKSTLRSKRFSRVNRVSYSPVLPRAWTNESRTVSSLFQDDLDSNSD 1110
CARMIL          ------------------------------------------------------------

CARMIL-GAP      SSGPSL                                                       1115
```

Journal of Cell Science • Supplementary information

Fig. S1. Alignment of the sequences of CARMIL and CARMIL-GAP. Sequences corresponding to the domains shared between CARMIL-GAP and CARMIL, and the GAP domain specific to CARMIL-GAP, are indicated by a colored highlighting that matches the color of the domains in the Figure 1 cartoon. Lines indicate identity, colons indicate highly conservative substitutions, and dots indicate moderately conservative substitutions. The sequence within the proline-rich domain of CARMIL-GAP that is underlined (residues 920 to 923) marks what may be a PXXP-based binding site for the SH3 domain of myosin 1 (Jung et al., 2001), although we did not investigate their possible interaction. Of note, it is unclear whether CARMIL-GAP has a bona fide HD domain based on this alignment. CARMIL-GAP is currently named gacW in dictyBase (DDB_G0290439; (Fey et al., 2013) and in Uniprot (Q54G18). Searches of the *Dictyostelium* genome for proteins other than CARMIL and CARMIL-GAP that contain a CPI domain identified only one other protein (the *Dictyostelium* homolog of the WASH complex subunit FAM21). Searches of the *Dictyostelium* genome also identified a second CARMIL-GAP gene (XP-003280987) that is very similar to CARMIL-GAP throughout except at the center of the CPI domain, where key residues, including the invariant arginine, are missing. Finally, searches of the mouse genome using two regions of sequence that are highly conserved between vertebrate CARMIL-1, CARMIL-2 and CARMIL-3 did not identify any CARMIL-like proteins that contain a GAP domain, arguing that the regulation of Rho-related GTPases by vertebrate CARMIL proteins (Stark et al., 2017) is restricted to regulation in trans.

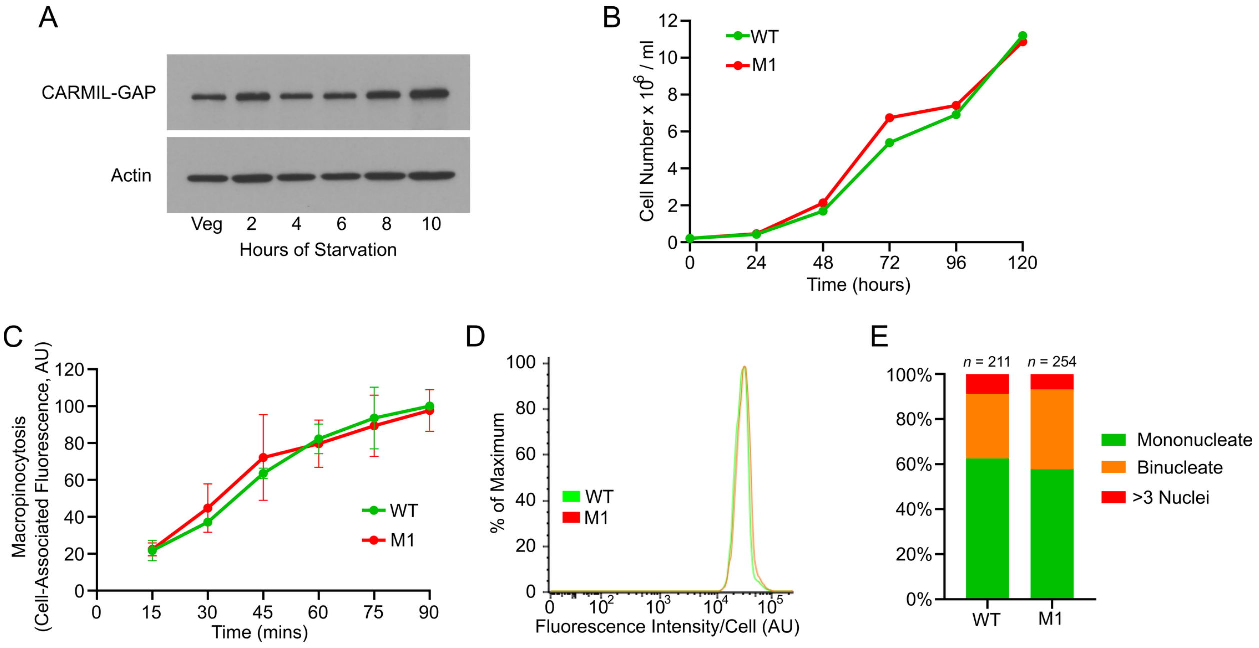

Fig. S2. Vegetative CARMIL-GAP null cells do not exhibit defects in growth rate, macropinocytosis, F-actin content, or cytokinesis. (A) Western blot of whole cell extracts of vegetative AX3 cells (“Veg”) and AX3 cells starved for 2, 4, 6, 8, and 10 hours on black filters, probed with the anti-CARMIL-GAP antibody. (B) Growth curve for WT and MI KO cells. Consistent with this one example, routine maintenance of these cells side by side did not reveal any obvious difference in growth rate. (C) Rate of macropinocytic uptake of FITC-labeled dextran by WT and M1 KO cells (WT 15 min, 21.7 ± 5.6 AU; WT 30 min, 37.1 ± 1.2 AU; WT 45 min, 63.5 ± 2.8 AU; WT 60 min, 82.2 ± 8.1 AU; WT 75 min, 98.5 ± 16.7 AU; WT 90 min, 100.0 ± 0.0 AU; M1 15 min, 22.3 ± 3.5 AU; M1 30 min, 44.8 ± 13.1 AU; M1 45 min, 72.1 ± 23.2 AU; M1 60 min, 79.6 ± 12.7 AU; M1 75 min, 89.3 ± 16.6 AU; WT 90 min, 97.6 ± 11.3 AU; N=3). (D) Total cellular F-actin content for WT and M1 KO cells, as determined by FACs analysis of cells stained with FITC-phalloidin (Jung et al., 2016). (E) Percent of mid-log WT and M1 KO cells with one (green), two (orange), or 3 or more (red) nuclei per cell. The total number of cells scored on three different days is indicated at the top of each bar.

Journal of Cell Science • Supplementary information

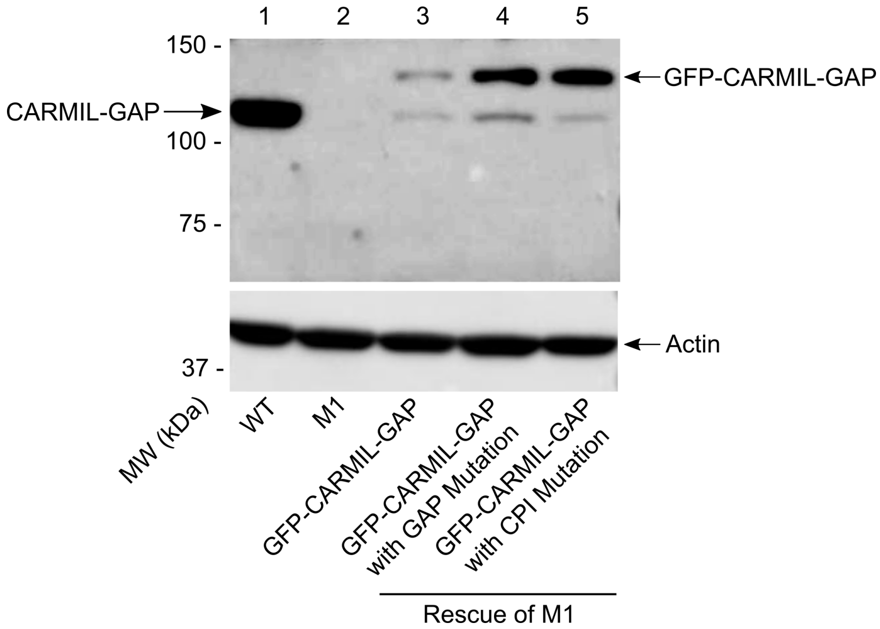

Fig. S3. Western blots of rescued CARMIL-GAP KO M1. Shown is a Western blot of whole cell extracts of WT cells (lane 1), CARMIL-GAP KO M1 (lane 2), and CARMIL-GAP KO M1 rescued with GFP-CARMIL-GAP (lane 3), GFP-CARMIL-GAP with the GAP mutation (lane 4), and GFP-CARMIL-GAP with the CPI mutation (lane 5), and probed with antibodies to CARMIL-GAP and actin as a loading control (of note, this single blot was cut into two pieces to probe with the two antibodies). The weak band in lanes 3-5 that corresponds in size to CARMIL-GAP most likely reflects the proteolytic cleavage of GFP from the GFP-CARMIL-GAP fusion proteins. The fact that M1 is fully rescued by very modest expression of GFP-CARMIL-GAP is consistent with our unpublished data on CARMIL, where CARMIL null cells are also fully rescued by very modest expression of GFP-CARMIL. Modest expression of GFP-tagged CARMIL and CARMIL-GAP may be sufficient to support the full rescue their null lines because the addition of GFP appears to favor the unfolded, active conformation of these proteins (Uruno et al 2006, Fujiwara et al, 2014, unpublished results).

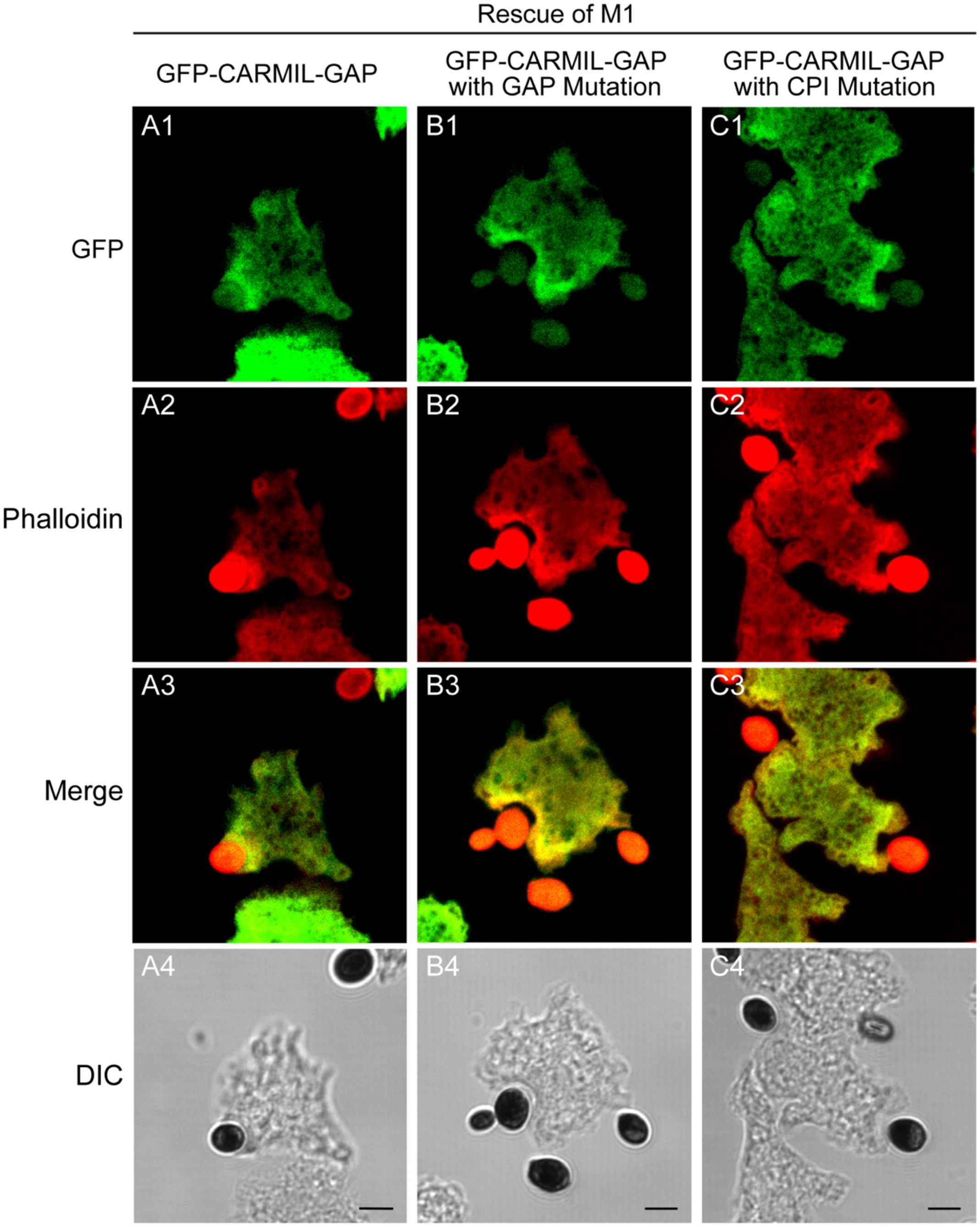

Fig. S4. GFP-CARMIL-GAP, GFP-CARMIL-GAP with the GAP mutation, and GFP-CARMIL-GAP with the CPI mutation localize to the phagocytic cup in rescued KO M1. Shown are representative examples of CARMIL-GAP KO M1 cells expressing GFP-CARMIL-GAP (A1-A4), GFP-CARMIL-GAP with the GAP mutation (B1-B4), and GFP-CARMIL-GAP with the CPI mutation (C1-C4) that were incubated with yeast particles as a phagocytic substrate and then fixed and stained with Phalloidin. The faint green signal on the yeast particles in Panels A1, B1 and C1 is due to bleed through from the extremely intense Phalloidin signal on these particles in the red channel. Mag bars: 1.6 µm.

Journal of Cell Science • Supplementary information

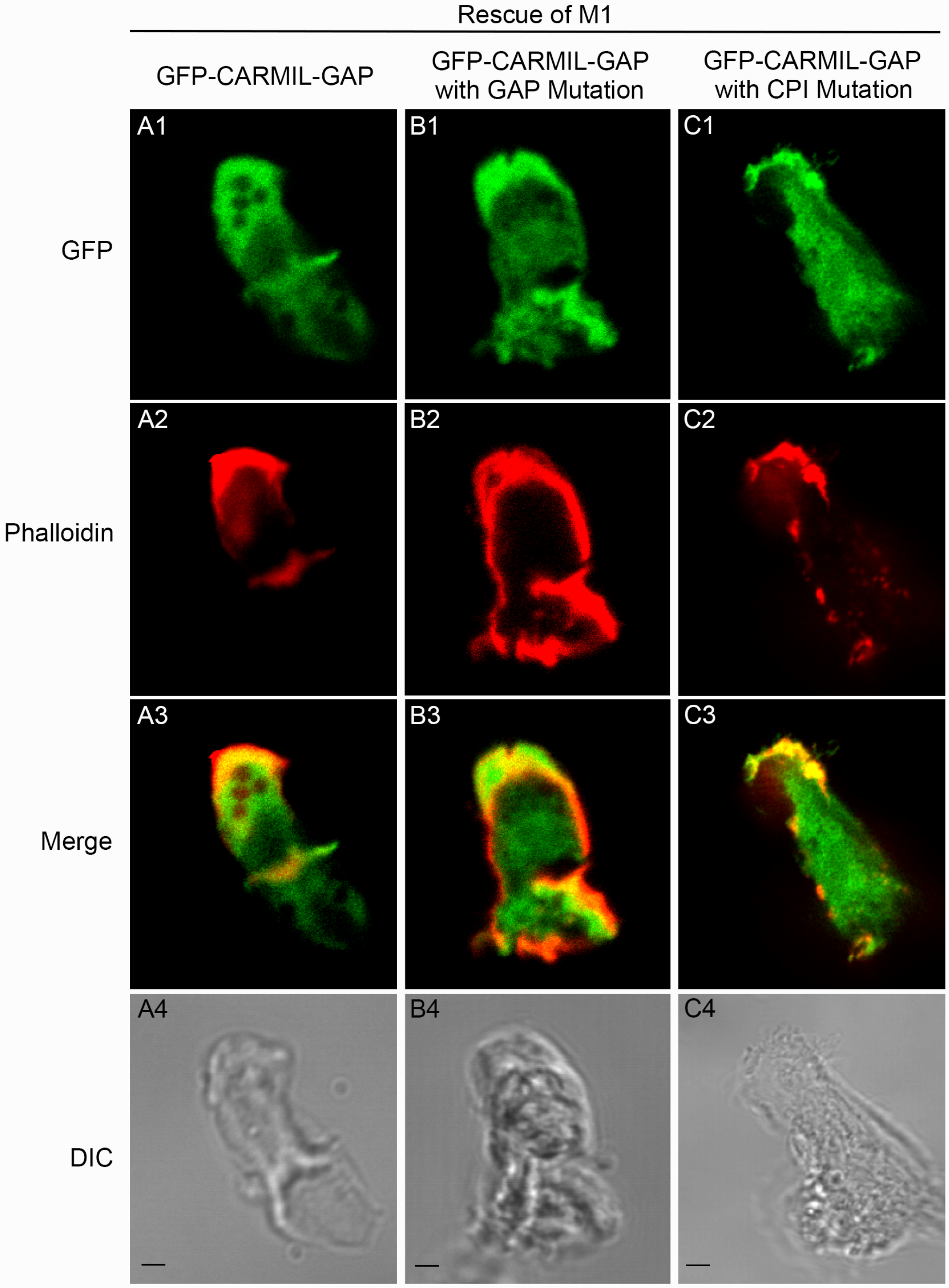

Fig. S5. GFP-CARMIL-GAP, GFP-CARMIL-GAP with the GAP mutation, and GFP-CARMIL-GAP with the CPI mutation localize to the leading edge in rescued, ripple-stage KO M1. Shown are representative examples of motile, ripple-stage CARMIL-GAP KO M1 cells expressing GFP-CARMIL-GAP (A1-A4), GFP-CARMIL-GAP with the GAP mutation (B1-B4), and GFP-CARMIL-GAP with the CPI mutation (C1-C4) that were fixed and stained with Phalloidin (the cells are aligned so that the hyaline zone at their leading edge (see the DIC image) is pointing up and slightly to the left). Mag bars: 3.4 µm.

Journal of Cell Science • Supplementary information

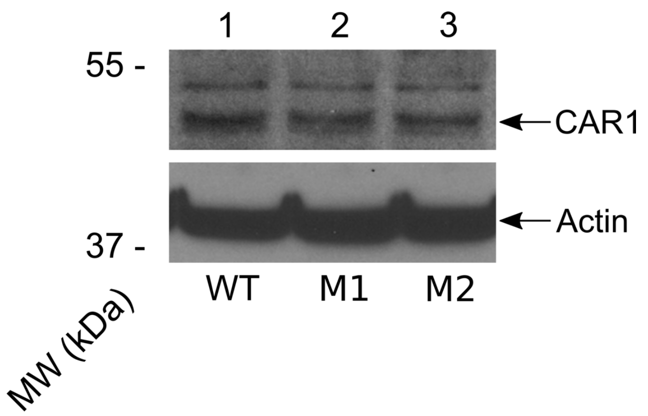

Fig. S6. CAR1 Western blot. Shown is a Western blot of whole cell extracts of ripple-stage WT cells (lane 1), CARMIL-GAP KO M1 (lane 2), and CARMIL-GAP KO M2 (lane 3) probed with antibodies to the cAMP receptor CAR1 and actin as a loading control (of note, this single blot was cut into two pieces to probe with the two antibodies). The blot is presented in such a way as to allow direct comparison with a similar blot published by Jung et al (2016), which included a vegetative cell extract to show that the CAR1 band at ~50 kDa is present only in starved cells, as expected.

Table S1. Curated list of proteins that bound to GST-GAP. List of proteins identified by mass spec in the eluate of the GST-GAP affinity column after removing ribosomal proteins, mitochondrial matrix proteins, proteins related to transcription/translation, and any proteins identified by less than three peptides.

Click here to download Table S1

Journal of Cell Science • Supplementary information

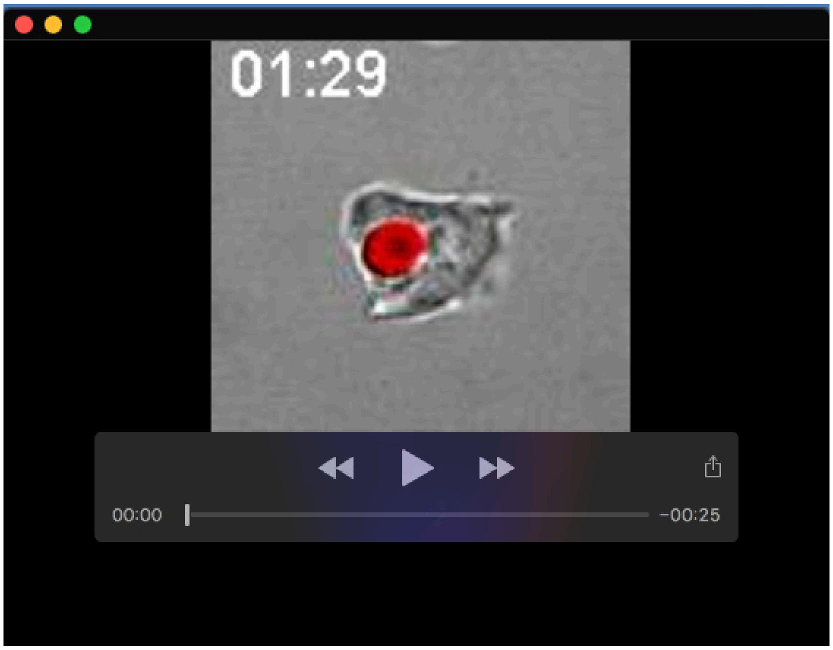

Movie 1. A failed yeast phagocytosis event by a WT cell. Images were taken every 5 sec for 2 min and are played back a 7 fps.

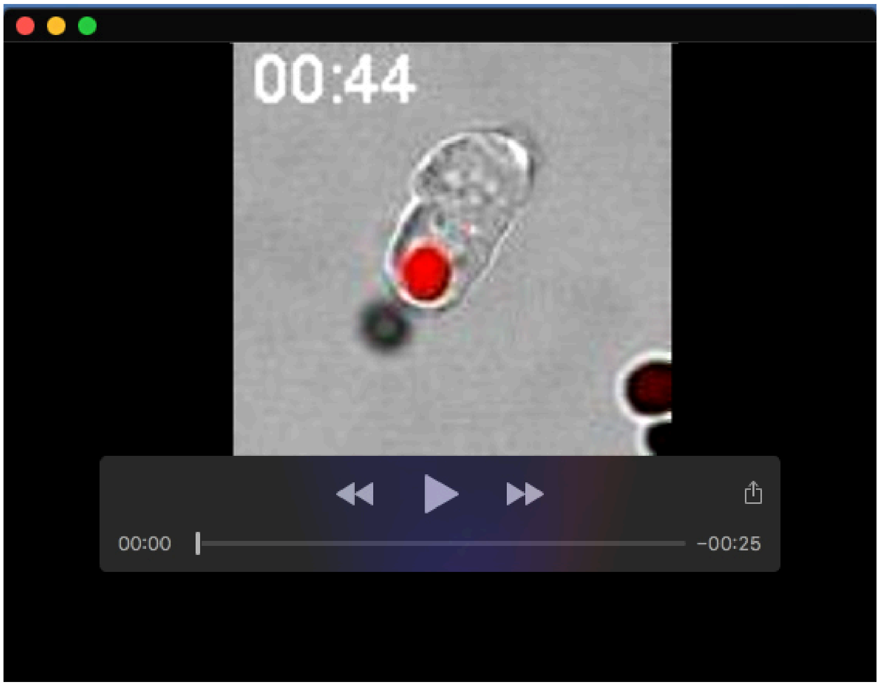

Movie 2. A successful yeast phagocytosis event by a M1 KO cell. Images were taken every 5 sec for 2 min and are played back a 7 fps.

Journal of Cell Science • Supplementary information

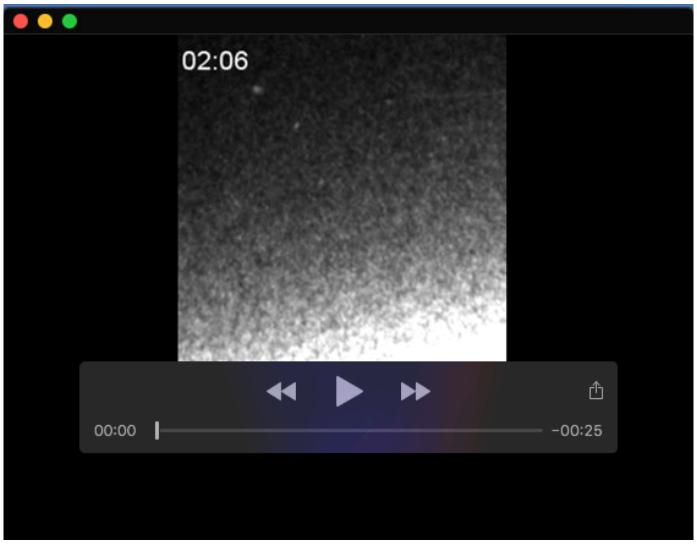

Movie 3. Steaming of WT cells. Images were taken every 10 min for 17 hrs and are played back a 30 fps.

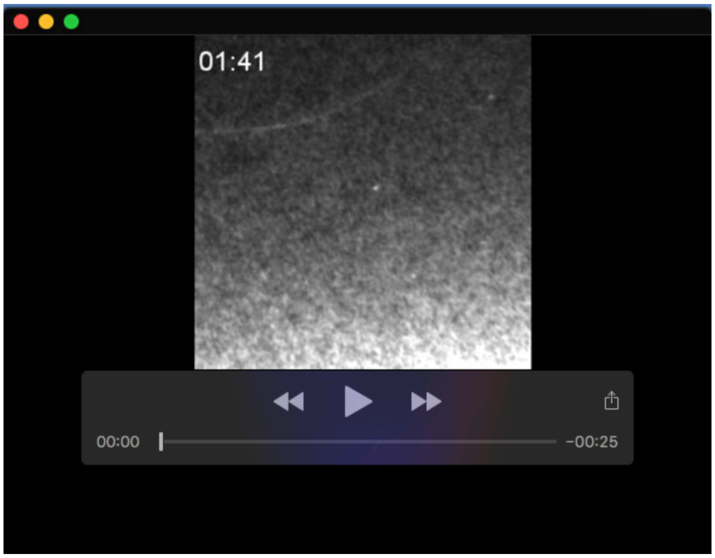

Movie 4. Steaming of M1 KO cells. Images were taken every 10 min for 17 hrs and are played back a 30 fps.

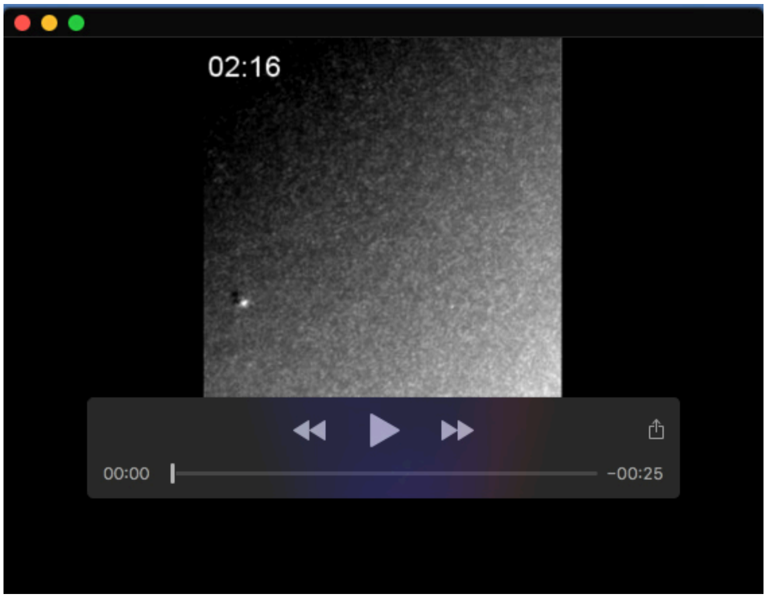

Movie 5. Steaming of M1 KO cells complemented with GFP-CARMIL-GAP. Images were taken every 10 min for 17 hrs and are played back a 30 fps.

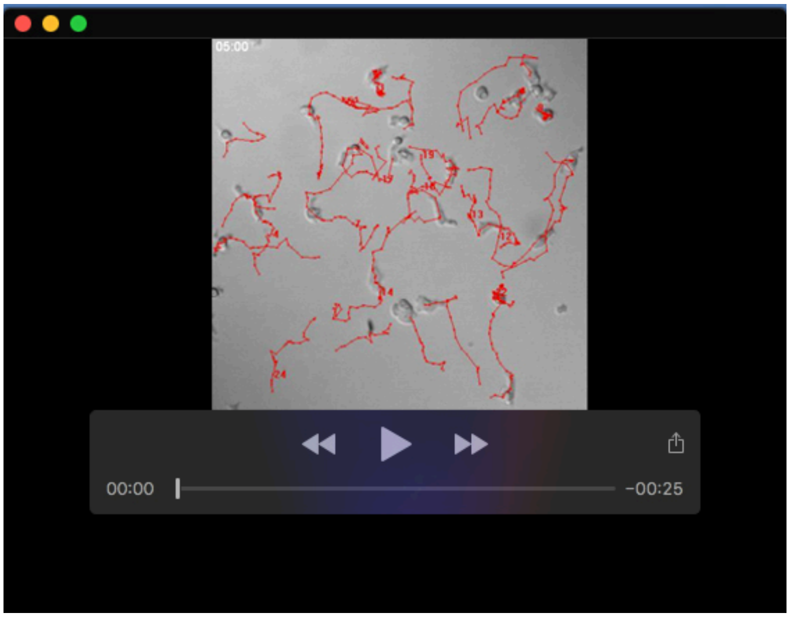

Movie 6. Path plot of the random motility of ripple-stage WT cells. Images were taken every 15 sec for 15 min and are played back at 30 fps.

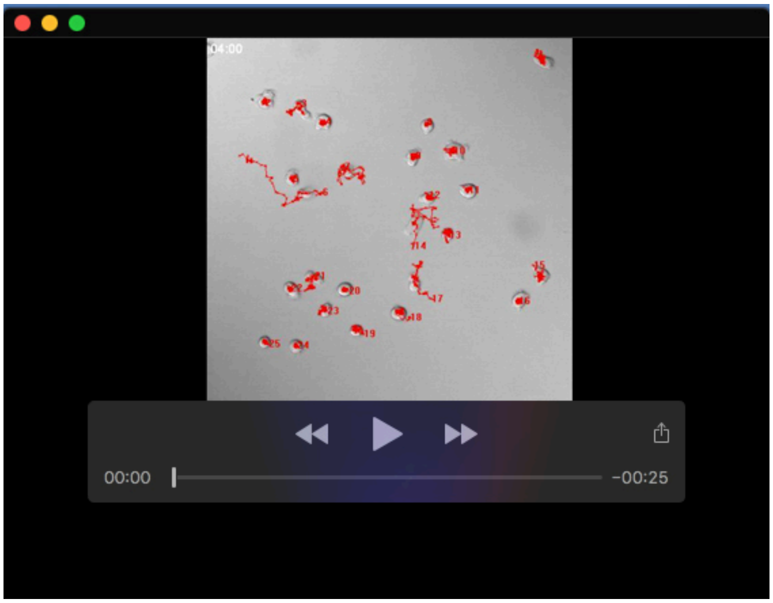

Movie 7. Path plot of the random motility of ripple-stage M1 KO cells. Images were taken every 15 sec for 15 min and are played back at 30 fps.

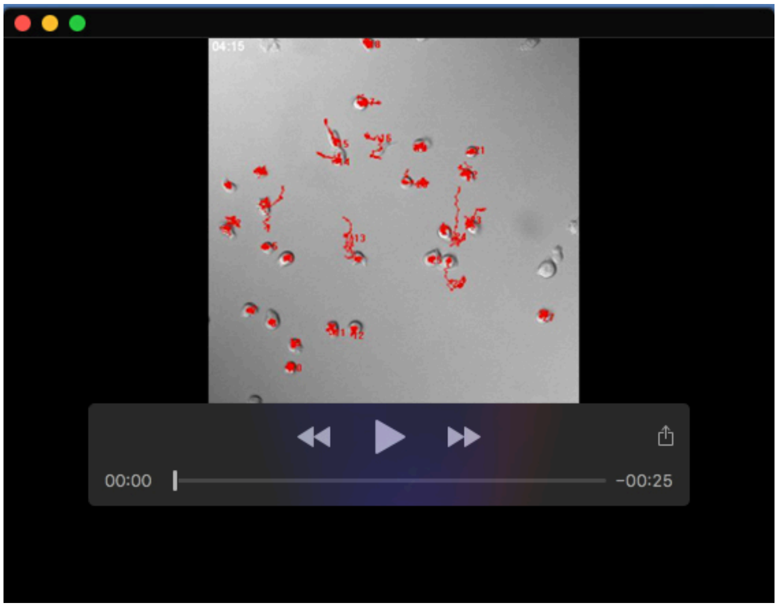

Movie 8. Path plot of the random motility of ripple-stage M2 KO cells. Images were taken every 15 sec for 15 min and are played back at 30 fps.

Journal of Cell Science • Supplementary information

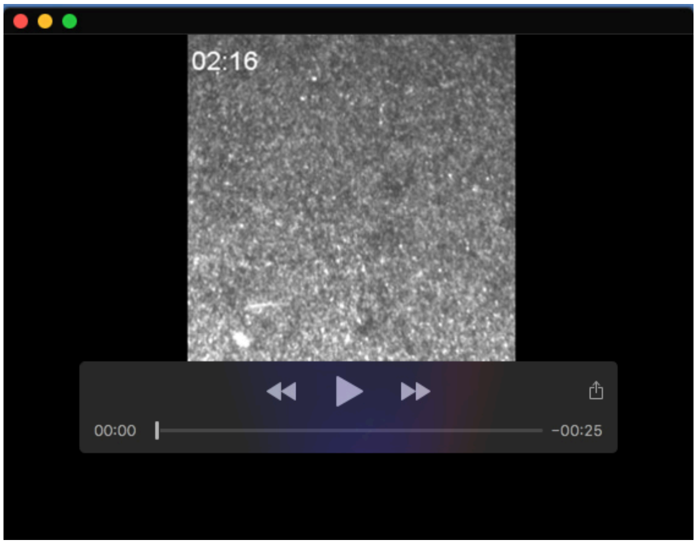

Movie 9. Steaming of M1 KO cells complemented with GFP-CARMIL-GAP with the GAP mutation. Images were taken every 10 min for 17 hrs and are played back a 30 fps.

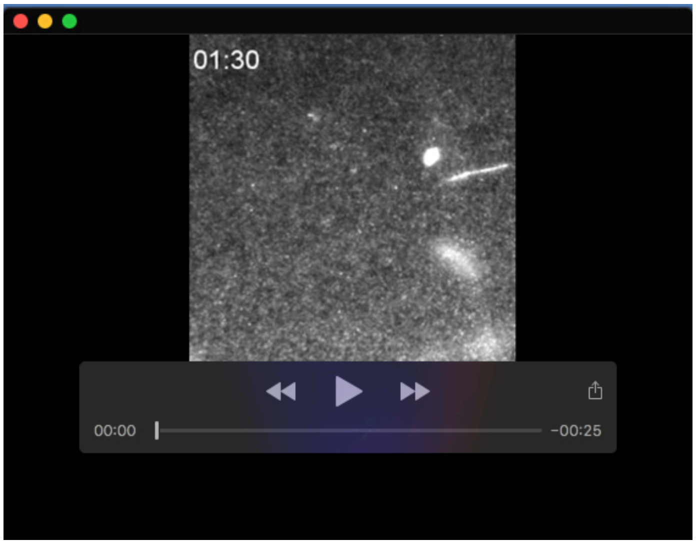

Movie 10. Steaming of M1 KO cells complemented with GFP-CARMIL-GAP with the CPI mutation. Images were taken every 10 min for 17 hrs and are played back a 30 fps.

Journal of Cell Science • Supplementary information
