# Supplementary material for: Dual regulation of the actin cytoskeleton by CARMIL-GAP
Source: J Cell Sci. 2022 Jun 20;135(12):jcs258704. doi: 10.1242/jcs.258704 (PMC9270954; doi:10.1242/jcs.258704)
Supplement: Reviewer comments [file joces-135-258704_review_history.pdf]

Journal of
Cell Science

Dual regulation of the actin cytoskeleton by CARMIL-GAP

Goeh Jung, Miao Pan, Christopher J. Alexander, Tian Jin and John A. Hammer
DOI: 10.1242/jcs.258704

Editor: Michael Way

Review timeline

Original submission:	27 March 2021
Editorial decision:	10 May 2021
First revision received:	22 March 2022
Editorial decision:	22 April 2022
Second revision received:	5 May 2022
Accepted:	9 May 2022

Original submission

First decision letter

MS ID#: JOCES/2021/258704

MS TITLE: Dual regulation of the actin cytoskeleton by CARMIL-GAP

AUTHORS: Goeh Jung, Miao Pan, Christopher J. Alexander, Tian Jin and John A. Hammer
ARTICLE TYPE: Research Article

We have now reached a decision on the above manuscript.

To see the reviewers' reports and a copy of this decision letter, please go to: https://submit-jcs.biologists.organd click on the 'Manuscripts with Decisions' queue in the Author Area. (Corresponding author only has access to reviews.)

As you will see, all three reviewers were positive but still raised a number of criticisms that prevent me from accepting the paper at this stage. They suggest, however, that a revised version might prove acceptable, if you can address their concerns with addition experiements and controls. In particular I think its important you show the localization of the two mutant proteins - ideally in live cells with an actin and/or membrane marker. A full description of the hits obtained by mass spec should be made available and all of the bar chart data should be plotted as individual points and statistical methods clearly described. If you think that you can deal satisfactorily with the criticisms on revision, I would be pleased to see a revised manuscript. We would then return it to the reviewers.

We are aware that you may be experiencing disruption to the normal running of your lab that makes experimental revisions challenging. If it would be helpful, we encourage you to contact us to discuss your revision in greater detail. Please send us a point-by-point response indicating where you are able to address concerns raised (either experimentally or by changes to the text) and where you will not be able to do so within the normal timeframe of a revision. We will then provide further guidance. Please also note that we are happy to extend revision timeframes as necessary.

Please ensure that you clearly highlight all text changes made in the revised manuscript in a different colour. Please avoid using 'Tracked changes' in Word files as these are lost in PDF conversion.

© 2022. Published by The Company of Biologists under the terms of the Creative Commons Attribution License (https://creativecommons.org/licenses/by/4.0/).

I should be grateful if you would also provide a point-by-point response detailing how you have dealt with the points raised by the reviewers in the 'Response to Reviewers' box. Please attend to all of the reviewers' comments. If you do not agree with any of their criticisms or suggestions please explain clearly why this is so.

Reviewer 1

Advance summary and potential significance to field

Jung and Hammer et al. have previously reported that CARMIL regulates actin dynamics as a scaffold for the assembly of the complex containing class I myosin, Arp2/3 complex, and actin capping proteins in Dictyostelium cells (PMID: 11425877). In the current manuscript, the authors identified the second CARMIL gene (termed CARMIL-GAP) in the Dictyostelium genome. Unlike the first CARMIL, CARMIL-GAP contains a potential RHO GAP sequence. By creating KO cells, the authors show that CARMIL-GAP functions in phagocytosis and chemotactic streaming. This work is carefully done, and I have only minor critiques as described below.

Comments for the author

1. It has been shown that the CARMIL interacts with regulators for RHO GTPases such as GFFs and GAPs in other systems (PMC5491179). Is the CARMIL-GAP in Dictyostelium only one in the CRAMIL family that contains GAP (or GEF) sequence?
2. The peptides data from mass spectrometry is not clear to me. Are only 13 peptides of RAC1 detected by mass spec and no other proteins were identified (very unlikely)? Is Rac1 (a) the only RHO GTPase detected by this mass spec? More comprehensive data presentation and clarification are necessary.
3. GST-CPI and GST-CPI RΔE should be included in Fig. 2D.
4. The bar graphs in Fig. 2C, Fig. 3C, and Fig. 7B and C should be shown as bars with individual data plots.
5. Fig. 3A needs actin as a loading control.
6. Fig. 3B should contain the size bar. The Y-axis of Fig. 3C (currently presented in pixel numer) should be presented in actual size (millimeter) or values relative to a control.
7. Amounts of GFP-CARMIL-GAP and its mutants should be tested by Western blotting. This is especially important to support the authors' conclusion that GAP activity is more critical than cap protein regulation in phagocytosis (Fig.4C) and chemotactic streaming (Fig. 6).
8. The authors describe that GFP-CARMIL-GAP is fully functional. Are they localized similarly to endogenous protein seen by IF at the phagocytotic cup and the leading edge of migrating cells? It is also interesting to see the localization of GAP and CPI mutants.
9. There is a typo in the Fig. 4 legend: (D) Shown are representative images of five cell samples quantified in Panel D. This should be Panel C.
10. The CARMIL-GAP already has a name and identification number. It is helpful to include the information for the reader to easily find the CARMIL-GAP gene in the existing data base such as Uniprot. https://www.uniprot.org/uniprot/Q54G18

Reviewer 2

Advance summary and potential significance to field

This is a well-written manuscript, characterising a new CARMIL orthologue in DIctyostelium, which uniquely contains an additional GAP domain. As CARMIL family proteins are major actin regulators though inhibiting Capping Protein dynamics, this provides a novel mechanism to coordinate small GTPase activation and actin filament dynamics.

The experiments described are logical, generally well-controlled, and make a convincing argument for a role of CARMIL-GAP in phagocytosis and migration in Dictyostelium. This is not particularly novel as it mirrors previous observations from the same group with cells defective in the canonical CARMIL gene, however they take this further by performing rescue experiments using versions of CARMIL-GAP specifically deficient in either GAP or Capping Protein regulatory activity. This clearly

© 2022. Published by The Company of Biologists under the terms of the Creative Commons Attribution License (https://creativecommons.org/licenses/by/4.0/).

demonstrates important functional roles for both functions. Whilst it seems that no equivalent protein exists outside the amoebazoa, this adds to the growing evidence that the coordinated regulation of GTPases and capping protein are of general importance.

I have a few fairly minor technical concerns that should be addressed, but overall their conclusions are sound, internally consistent, and fully justified by the data presented.

Comments for the author

Major points
It is very interesting that the GAP and Capping Protein interaction (CPI) domains are both important. However, their experiments rescuing with mutations in each domain assume the mutations work independently. It is possible that one or both interactions are required for the proper localisation of CARMIL-GAP. For example, if the interaction between the GAP domain and Rac is required to recruit CARMIL-GAP correctly, this would explain why the GAP mutation rescues less well and appears more important. To support their conclusion that the GAP domain is more important and add additional mechanistic insight, the authors should confirm how each rescue protein localises, ideally in live cells.
In figure 5 they show effects of CARMIL-GAP deletion on the ability to phagocytose yeast. They report that phagocytosis often fails and estimate from timelapses how long the yeast are bound before release. This is very hard to do from wide-field microscopy, and it is unclear how they determine when the yeast has been recognised, and when it is "released" vs just sitting near the cell surface. I therefore find panel 5A unconvincing. Do they propose the mutants have defects in recognising the yeast, enwrapping it, or internalising it?
In figures 6 and 7 they show that loss of CARMIL-GAP affects both streaming and random migration of starved Dictyostelium cells. However both assays are potentially confounded by potential defects in the initiation or progression of development. This is particularly true for the degree of polarised morphology they describe in figure 7C. They should therefore either confirm that the initiation of development is not disrupted (e.g. by Westen blotting for markers of development such as cAR1) and/or (better), measure vegetative cell motility and chemotaxis to folate where cells will be in the same state.
Whilst it is nice that they often show data points for individual cells where appropriate (E.g. 4C and 5A). It appears that statistics were performed using each cell as a biological repeat, rather than the independent experiments. This therefore uses a much enlarged n-number and an inaccurate statistical power (see detailed explanation in Lord er al J Cell Biol (2020) 219 (6): e202001064. https://doi.org/10.1083/jcb.202001064)
It is unlikely to change their conclusions, but they should replot these graphs appropriately. I also note that the statistical analysis section of their methods simply refers back to one of their previous articles. This is not sufficient and they should clearly describe how each value and statistical test (including the test type) was performed in the article -ideally in the figure legends.

Minor points
The alignment of the GAP domain in figure 1B is not particularly convincing. It could be improved by including additional GAP domains, perhaps including some from Dictyostelium, to better highlight the crucial, conserved residues.
I found the current structure a bit confusing as the figure panels are not always called out in sequence. For example, Figure panel 1C is only referred to after Figure 2, and the rescue experiments with the GAP and CPI mutations included in figures 4-7 are not referred to until the very end. I understand why the authors have done this, but would suggest either mentioning these rescues at the time, or putting them all together at the end to improve clarity.
The correct annotation for the Dictyostelium Rac isoforms is e.g. Rac1A etc (not Rac1a)

Reviewer 3

Advance summary and potential significance to field

Cytoskeletal dynamics are tightly regulated by a suite of actin binding proteins, small G-proteins regulatory GAPs and GEFs and downstream effectors of small G proteins. CARMILs are a family of actin regulators that act by inhibiting capping protein (CP) and are implicated in regulating signaling pathways.

© 2022. Published by The Company of Biologists under the terms of the Creative Commons Attribution License (https://creativecommons.org/licenses/by/4.0/).

Jung et al report the discovery of a second CARMIL family member in the amoeba Dictyostelium that harbors a GAP domain in its C-terminal homodimerization domain and establish its role in phagocytosis and cell migration. Interestingly, the GAP domain interacts with Rac1a, b and c and appears to be essential for CARMIL-GAP in vivo functions while the contribution of the CPI domain is not as significant. The work provides further support for CARMILs playing roles beyond regulating CP activity to include directly acting as regulators of signaling pathways.

Comments for the author

The results presented here are straightforward and convincingly establish an important role for CARMIL-GAP in phagocytosis and motility, but not pinocytosis or cytokinesis. The analysis of the phenotypes is clear and convincing, but they provide one with little understanding of how CARMIL-GAP is acting. While it would beyond the scope of this work to do a deep dive into mechanism, readers will still be left wondering. For example, the phagocytosis data suggest that the null mutants fail to trigger phagocytic cup formation and the motility phenotype suggests that there are defects in leading edge formation. No information is presented about the actin cytoskeleton in the mutants or any description of what the leading edge looks like (something that should be straightforward to assess with data in hand). The streaming defect is quite strong and it is likely that it is not simply due to the reduced motility (see below). If it were possible to provide some more details on this phenotype relevant for motility and/or signaling processes that must occur during streaming this would help one to better understand this phenotype.

Additional comments -
1) The authors show that the GAP domain binds mainly to GTP-Rac1a strongly suggesting that it could be acting, at least in part, to control Rac1a (and b,c) activity. However, in discussing the null mutant phenotypes the authors do not link them to what is known about cellular functions of Rac1a.
2) The different plots quite clearly show differences between the control and mutants. However, numerical values are in the legend, making this information less accessible to readers. The numerical data should be presented in a Table where they could be more readily compared.
3) The streaming defect is a bit puzzling. Other Dictyostelium mutants with similarly reduced speeds do have streaming defects, such as the MyoIB mutant, that but they are mainly delayed by several hours and not incapable of streaming as is shown here. What can account for the observed phenotyp here?
Also, in spite of a lack of streaming the mutants appear to form small aggregates - do they go any further in the developmental cycle to form any type of multicellular structure?
4) CARMIL-GAP appears to be associated with the cytoskeleton and not exclusively with the membrane (Fig 7D). The authors speculate that the GAP domain could play a role in localization through its interaction with Rac1a. Are the GAP or CPI deletion mutants mislocalized?
5) Is CARMIL-GAP unique to Dictyostelium and related species? The authors should briefly comment on this.
6) The statistical analysis is said to be described in an earlier paper, Jung et al, 2016 (PNAS vol 113 - the citation is incomplete) yet that paper does not present any information on statistical analysis in the Methods section. Perhaps one can decipher what was done by going through the paper and figures, but this should not be necessary. It is highly preferable that the statistical analysis be described in the Methods. The number of samples and biological replicates should be reported.

Minor points -
a) The gene encoding CARMIL-GAP is currently named gacW (GTPase activator for racC) in Dictybase. The authors should be encouraged to propose a more appropriate gene name to the Dictybase curators.
b) There is a straight line at the end of the box diagram of CARMIL-GAP in Fig. 1A. Please describe what this indicates.
c) CARMIL was originally identified as a Myosin 1 binding protein, does CARMIL-GAP have a binding site for the SH3 domain of Myosin 1 similar to CARMIL?
d) When describing the complementation experiments that authors say that they 're-express' a GFP fusion of CARMIL-GAP. Strictly speaking they are introducing a GFP- CARMIL-GAP to rescue the null defect.
e) The images of streaming are quite dark. Is it possible to make them a bit lighter to see the whole field a bit better?

© 2022. Published by The Company of Biologists under the terms of the Creative Commons Attribution License (https://creativecommons.org/licenses/by/4.0/).

f) The Discussion states that "Rac1a is expressed at vastly higher levels than Rab1b and Rac1c" and cites Fey et al 2013 (a Methods paper?). The basis for this statement should be explained, presumably the authors are referring to RNA expression levels.

First revision

Author response to reviewers' comments

Reviewer 1 Advance Summary and Potential Significance to Field:
Jung and Hammer et al. have previously reported that CARMIL regulates actin dynamics as a scaffold for the assembly of the complex containing class I myosin, Arp2/3 complex, and actin capping proteins in Dictyostelium cells (PMID: 11425877). In the current manuscript, the authors identified the second CARMIL gene (termed CARMIL-GAP) in the Dictyostelium genome. Unlike the first CARMIL, CARMIL-GAP contains a potential RHO GAP sequence. By creating KO cells, the authors show that CARMIL-GAP functions in phagocytosis and chemotactic streaming. This work is carefully done, and I have only minor critiques as described below.

Reviewer 1 Comments for the Author:

1. It has been shown that the CARMIL interacts with regulators for RHO GTPases such as GFFs and GAPs in other systems (PMC5491179). Is the CARMIL-GAP in Dictyostelium only one in the CARMIL family that contains GAP (or GEF) sequence?

We thank the reviewer for their question and added the following information in the legend to Figure S1: “Finally, searches of the *Dictyostelium* genome also identified a second CARMIL-GAP gene (XP-003280987) that is very similar to CARMIL-GAP throughout except at the center of the CPI domain, where key residues, including the invariant arginine, are missing.”

2. The peptides data from mass spectrometry is not clear to me. Are only 13 peptides of RAC1 detected by mass spec and no other proteins were identified (very unlikely)? Is Rac1 (a) the only RHO GTPase detected by this mass spec? More comprehensive data presentation and clarification are necessary.

We agree completely with Reviewer 1 and have now included all of our mass spec data in Table S1, together with additional text in the Results and Discussion sections of the paper. As detailed in the legend to Table S1, our raw mass spec results contained 364 proteins that were represented by three or more peptides. After removing mitochondrial proteins, ribosomal proteins and transcription factors, we were left with a list of 84 proteins, which are shown in Table S1. We then revised the text in the Results to read as follows: “In terms of Rho-related GTPases, the curated list of bound proteins (Table S1; see the legend for details) contained only two Rho-related GTPases: the Rac-like GTPase RacE (10 distinct peptides) and Rac1 (6 distinct peptides; Figure 2, Panel A). With regard to Rac1, while peptides 1, 3 and 4 are present in all three Rac1 isoforms (Rac1a, Rac1b, and Rac1c), and peptide 5 is present in both Rac1a and Rac1b, peptides 2 and 6 are present only in Rac1a. These results, together with the fact that Rac1a is expressed at vastly higher levels than Rac1b and Rac1c in both vegetative and starved cells based on RNA measurements (available on dictyExpress; Stajdohar et al., 2015), argue that CARMIL-GAP’s GAP domain interacts preferentially within cells with the 1a isoform of *Dictyostelium* Rac1. Given the phenotype of CARMIL-GAP null cells (see below), which do not exhibit a defect in cytokinesis when grown in suspension (a process regulated by RacE; reviewed in Rivero and Xiong, 2016), but do exhibit a pronounced defect in phagocytosis (a process regulated by Rac1a; reviewed in Rivero and Xiong, 2016), we focused on the role of CARMIL-GAP’s GAP domain in regulating the nucleotide state of Rac1a.”

3. GST-CPI and GST-CPI RΔE should be included in Fig. 2D.

We thank Reviewer 1 and have now included a Coomassie Blue-stained gel of these two GST fusion proteins in Figure 2, Panel D.

© 2022. Published by The Company of Biologists under the terms of the Creative Commons Attribution License (https://creativecommons.org/licenses/by/4.0/).

4. The bar graphs in Fig. 2C, Fig. 3C, and Fig. 7B and C should be shown as bars with individual data plots.

We thank Reviewer 1 and have now added the individual data points in these four panels.

5. Fig. 3A needs actin as a loading control.

We thank Reviewer 1 for pointing this out and have addressed this by modifying the legend to Figure 3, Panel A, as follows: “(A) Western blot of whole cell extracts prepared from equal numbers of control AX3 cells (WT) and CARMIL-GAP null cell lines M1 and M2 probed with an antibody to CARMIL-GAP. The cross reacting band at ~63 kDa (see arrowhead) serves as a loading control”. In addition, new Figure S3 presents a Western blot of whole cell extracts for WT cells and the M1 KO that includes an actin loading control. This is also mentioned in the legend to Figure 3, Panel A, as follows: “(see also Figure S3, lanes 1 and 2, which included an actin loading control)”.

6. Fig. 3B should contain the size bar. The Y-axis of Fig. 3C (currently presented in pixel number) should be presented in actual size (millimeter) or values relative to a control.

We thank Reviewer 1 for these suggestions and have added a size bar to Panel B and converted the Y axis in Panel C from pixels to mm^2.

7. Amounts of GFP-CARMIL-GAP and its mutants should be tested by Western blotting. This is especially important to support the authors' conclusion that GAP activity is more critical than cap protein regulation in phagocytosis (Fig.4C) and chemotactic streaming (Fig. 6).

We thank Reviewer 1 for raising this important issue, which we agree requires the addition of Western blots of M1 null cells rescued with GFP-CARMIL-GAP, GFP-CARMIL-GAP with the GAP mutation, and GFP-CARMIL-CAP with the CPI mutation. New Figure S3 shows that these two mutant proteins are expressed in complemented M1 null cells at levels exceeding that of wild type GFP-CARMIL-GAP in complemented M1 null cells, which exhibit complete rescue. This result argues that the inability of the GAP mutant to rescue either phagocytosis or streaming, and the inability of the CPI mutant to fully rescue these behaviors, cannot be attributed to insufficient expression.

8. The authors describe that GFP-CARMIL-GAP is fully functional. Are they localized similarly to endogenous protein seen by IF at the phagocytotic cup and the leading edge of migrating cells? It is also interesting to see the localization of GAP and CPI mutants.

We thank Reviewer 1 for raising this important issue, which we agree requires the addition of localization data for M1 null cells rescued with GFP-CARMIL-GAP, GFP-CARMIL-GAP with the GAP mutation, and GFP-CARMIL-CAP with the CPI mutation. New Figures S4 and S5 show that all three GFP tagged proteins localize to phagocytic cups (Figure S4) and to the leading edge of crawling, ripple-stage cells (Figure S5) in complemented M1 null cells. These results argue that the inability of the GAP mutant to rescue either phagocytosis or streaming, and the inability of the CPI mutant to fully rescue these behaviors, cannot be attributed to mis localization.

9. There is a typo in the Fig. 4 legend: (D) Shown are representative images of five cell samples quantified in Panel D. This should be Panel C.

Corrected. Thank you.

10. The CARMIL-GAP already has a name and identification number. It is helpful to include the information for the reader to easily find the CARMIL-GAP gene in the existing data base such as Uniprot.

https://www.uniprot.org/uniprot/Q54G18. We thank Reviewer 1 for this suggestion and now provide this information in the Legend to Figure S1 as follows “CARMIL-GAP is currently named gacW in dictyBase (DDB_G0290439; Fey et al., 2013) and in Uniprot (Q54G18).”

© 2022. Published by The Company of Biologists under the terms of the Creative Commons Attribution License (https://creativecommons.org/licenses/by/4.0/).

Reviewer 2 Advance Summary and Potential Significance to Field:
This is a well-written manuscript, characterising a new CARMIL orthologue in DIctyostelium, which uniquely contains an additional GAP domain. As CARMIL family proteins are major actin regulators though inhibiting Capping Protein dynamics, this provides a novel mechanism to coordinate small GTPase activation and actin filament dynamics.

The experiments described are logical, generally well-controlled, and make a convincing argument for a role of CARMIL-GAP in phagocytosis and migration in Dictyostelium. This is not particularly novel as it mirrors previous observations from the same group with cells defective in the canonical CARMIL gene, however they take this further by performing rescue experiments using versions of CARMIL-GAP specifically deficient in either GAP or Capping Protein regulatory activity. This clearly demonstrates important functional roles for both functions. Whilst it seems that no equivalent protein exists outside the amoebazoa, this adds to the growing evidence that the coordinated regulation of GTPases and capping protein are of general importance.

I have a few fairly minor technical concerns that should be addressed, but overall their conclusions are sound, internally consistent, and fully justified by the data presented.

Reviewer 2 Comments for the Author:

Major points

It is very interesting that the GAP and Capping Protein interaction (CPI) domains are both important. However, their experiments rescuing with mutations in each domain assume the mutations work independently. It is possible that one or both interactions are required for the proper localisation of CARMIL-GAP. For example, if the interaction between the GAP domain and Rac is required to recruit CARMIL-GAP correctly, this would explain why the GAP mutation rescues less well and appears more important. To support their conclusion that the GAP domain is more important and add additional mechanistic insight, the authors should confirm how each rescue protein localises, ideally in live cells.

We thank Reviewer 2 for raising this important issue, which is similar to a concern raised by Reviewer 1. New Figures S4 and S5 show that GFP-CARMIL-GAP, GFP-CARMIL-GAP with the GAP mutation, and GFP-CARMIL-CAP with the CPI mutation all localize to phagocytic cups (Figure S4) and to the leading edge of crawling, ripple-stage cells (Figure S5) in complemented M1 null cells. These results argue that CARMIL-GAP's localization to these sites does not require a functioning GAP domain or a functioning CPI domain. They also argue that the inability of the GAP mutant to rescue either phagocytosis or streaming, and the inability of the CPI mutant to fully rescue these behaviors, cannot be attributed to mis localization. Given this, and given the new Western blot data in Figure S3 (see our response to Reviewer 1, Question 7), we conclude that the inability of the GAP mutant to rescue either phagocytosis or streaming, and the inability of the CPI mutant to fully rescue these behaviors, is due to their inability to regulate Rac1a and CP, respectively, rather than to mis localization or insufficient expression.

In figure 5 they show effects of CARMIL-GAP deletion on the ability to phagocytose yeast. They report that phagocytosis often fails and estimate from time lapses how long the yeast are bound before release. This is very hard to do from wide-field microscopy, and it is unclear how they determine when the yeast has been recognized, and when it is "released" vs just sitting near the cell surface. I therefore find panel 5A unconvincing. Do they propose the mutants have defects in recognising the yeast, enwrapping it, or internalising it?

We thank Reviewer 2 for their comment and agree that our method does not provide a "signal" (akin to a FRET signal for protein: protein interactions) for when the cell and the yeast are touching. That said, we think our time-lapse imaging, as described in the Methods and presented in the still images in Figure 5, Panels C1-C5 and D1-D5, and in Movies 1 and 2, do make clear when a yeast particle that is immediately adjacent to a *Dictyostelium* cell (i.e. in apparent contact) is either internalized or released/floats away. We also note that the result we obtained using our method- a 50% reduction in the yeast phagocytosis when CARMIL-GAP is missing- is in line with phagocytosis data we obtained using other methods and other phagocytic substrates (plastic beads and bacteria).

© 2022. Published by The Company of Biologists under the terms of the Creative Commons Attribution License (https://creativecommons.org/licenses/by/4.0/).

In figures 6 and 7 they show that loss of CARMIL-GAP affects both streaming and random migration of starved Dictyostelium cells. However both assays are potentially confounded by potential defects in the initiation or progression of development. This is particularly true for the degree of polarised morphology they describe in figure 7C. They should therefore either confirm that the initiation of development is not disrupted (e.g. by Westen blotting for markers of development such as cAR1) and/or (better), measure vegetative cell motility and chemotaxis to folate where cells will be in the same state.

We thank the reviewer for their comments, which we agree raise questions regarding the underlying cause of the fairly severe defect in streaming exhibited by CARMIL-GAP null cells. The fact that this defect is not rescued by complementation of null cells with a version of CARMIL-GAP containing a single point mutation that largely abrogates its GAP activity puts the focus squarely on this activity. While we interpreted the streaming phenotype solely in the context of the GAP-domain dependent regulation of Rac1a (and hence actin assembly/cell migration), we agree that this domain could be also be regulating GTPases that are required for the progression of *Dictyostelium's* developmental program. This possibility remains despite the fact that ripple-stage null cells express near normal levels of CAR1 (new Figure S6). To acknowledge the reviewers important point, we have added the following three sentences at the end of the first paragraph in the Discussion: "It is also important to note that while the discussion below focuses on the role of CARMIL-GAP's GAP domain in regulating Rac1a, we cannot exclude the possibility that this domain regulates additional Rho-related GTPases (e.g. RacE), and that their mis regulation contributes to the defects in actin-dependent processes exhibited by CARMIL-GAP null cells. In a similar vein, we cannot exclude the possibility that the profound defect in streaming exhibited by cells lacking CARMIL-GAP's GAP activity is due at least in part to the mis regulation of GTPases required for progression of *Dictyostelium's* developmental program. The pronounced defect in phagocytosis exhibited by null cells cannot be attributed, however, to defects in this developmental program." Finally, with regard to measuring chemotaxis to folate, the first author of this study (Goeh Jung), who is responsible for most of the work in it, and who is the only person in the Hammer lab to have ever worked with *Dictyostelium*, retired for health reasons in November of 2020. While he was able to return to the lab for a limited amount of time in the fall of 2021 to do experiments, he was only able address those experiments listed by the editor as essential for acceptance, which did not include measuring chemotaxis to folate.

Whilst it is nice that they often show data points for individual cells where appropriate (E.g. 4C and 5A). It appears that statistics were performed using each cell as a biological repeat, rather than the independent experiments. This therefore uses a much enlarged n-number and an inaccurate statistical power (see detailed explanation in Lord er al J Cell Biol (2020) 219 (6): e202001064. https://doi.org/10.1083/jcb.202001064). It is unlikely to change their conclusions, but they should replot these graphs appropriately. I also note that the statistical analysis section of their methods simply refers back to one of their previous articles. This is not sufficient and they should clearly describe how each value and statistical test (including the test type) was performed in the article - ideally in the figure legends.

We thank the reviewer for their comment and agree with them that it is important to present means and SDs of measurements on individual cells, as stable *Dictyostelium* cell lines derived from single cells (like we did here) typically exhibit significant cell-to-cell variation in expression level. That said, we agree that some of our figures should provide an indication of the experiment-to-experiment variability by including the experimental means. Revised Figures 3C (plaque size data), 4C (microscope-based phagocytosis data), 7B (speed of ripple-stage cells), and 7C (roundness of ripple-stage cells) now include red, green and blue squares indicating the means of the three independent experiments. In essentially every case, the three squares fall quite close to one another and very close to the mean for the individual cell measurements.
The only modest exception is one experiment in Figure 4C, where the mean for WT cells in one experiment (indicated by the red square) is somewhat lower than the other two experimental means (although still well within the deviation for the individual cells). Finally, we have now included how we performed statistical analyses in the revised Methods.

© 2022. Published by The Company of Biologists under the terms of the Creative Commons Attribution License (https://creativecommons.org/licenses/by/4.0/).

Minor points

The alignment of the GAP domain in figure 1B is not particularly convincing. It could be improved by including additional GAP domains, perhaps including some from Dictyostelium, to better highlight the crucial, conserved residues.

We thank Reviewer 2 for their comment and have now included another GAP domain in the sequence alignment presented in Figure 1, Panel B.

I found the current structure a bit confusing as the figure panels are not always called out in sequence. For example, Figure panel 1C is only referred to after Figure 2, and the rescue experiments with the GAP and CPI mutations included in figures 4-7 are not referred to until the very end. I understand why the authors have done this, but would suggest either mentioning these rescues at the time, or putting them all together at the end to improve clarity.

We thank Reviewer 2 for their comment but would prefer to keep all the figure panels containing gels and biochemical results in Figure 2. We hope this is acceptable. We would also prefer to present the rescue experiments with the GAP and CPI mutants at the end of the Results as that data represents our final dissection of CARMIL-GAP function. We hope this is acceptable.

The correct annotation for the Dictyostelium Rac isoforms is e.g. Rac1A etc (not Rac1a).

We thank Reviewer 2 for their comment. That said, the review on Rho signaling in *Dictyostelium* by Rivero and Xiong that we refer too often in our manuscript, and which to our knowledge is the best review on this subject, refers to the Rac1 isoforms as Rac1a, Rac1b, and Rac1c. We can of course change the letters to capitals if the reviewer insists.

Reviewer 3 Advance Summary and Potential Significance to Field:
Cytoskeletal dynamics are tightly regulated by a suite of actin binding proteins, small G- proteins, regulatory GAPs and GEFs and downstream effectors of small G proteins. CARMILs are a family of actin regulators that act by inhibiting capping protein (CP) and are implicated in regulating signaling pathways. Jung et al report the discovery of a second CARMIL family member in the amoeba Dictyostelium that harbors a GAP domain in its C-terminal homodimerization domain and establish its role in phagocytosis and cell migration.
Interestingly, the GAP domain interacts with Rac1a, b and c and appears to be essential for CARMIL-GAP in vivo functions while the contribution of the CPI domain is not as significant. The work provides further support for CARMILs playing roles beyond regulating CP activity to include directly acting as regulators of signaling pathways.

Reviewer 3 Comments for the Author:
The results presented here are straightforward and convincingly establish an important role for CARMIL-GAP in phagocytosis and motility, but not pinocytosis or cytokinesis. The analysis of the phenotypes is clear and convincing, but they provide one with little understanding of how CARMIL-GAP is acting. While it would beyond the scope of this work to do a deep dive into mechanism, readers will still be left wondering. For example, the phagocytosis data suggest that the null mutants fail to trigger phagocytic cup formation and the motility phenotype suggests that there are defects in leading edge formation. No information is presented about the actin cytoskeleton in the mutants or any description of what the leading edge looks like (something that should be straightforward to assess with data in hand). The streaming defect is quite strong and it is likely that it is not simply due to the reduced motility (see below). If it were possible to provide some more details on this phenotype relevant for motility and/or signaling processes that must occur during streaming this would help one to better understand this phenotype.

We thank Reviewer 3 for their comments and agree that more could be done to characterize the phenotypes we present. That said, we think we have presented a fairly thorough initial characterization of CARMIL-GAP function that includes a reasonable amount of mechanistic insight and ask that we be allowed to address requests for additional data at a later date.

© 2022. Published by The Company of Biologists under the terms of the Creative Commons Attribution License (https://creativecommons.org/licenses/by/4.0/).

Additional comments -

1) The authors show that the GAP domain binds mainly to GTP-Rac1a strongly suggesting that it could be acting, at least in part, to control Rac1a (and b,c) activity. However, in discussing the null mutant phenotypes the authors do not link them to what is known about cellular functions of Rac1a.

We thank Reviewer 3 for their comment and note that the third paragraph of the Discussion cited numerous papers linking Rac1a to cell migration and phagocytosis in *Dictyostelium*. That said, we now included several additional references along with the comprehensive review of Rho-GTPases signaling in *Dictyostelium* by Rivero and Xiong.

2) The different plots quite clearly show differences between the control and mutants. However, numerical values are in the legend, making this information less accessible to readers. The numerical data should be presented in a Table where they could be more readily compared. We thank Reviewer 3 for this suggestion and have moved the numerical values and statistical measures from the legends for Figure 3 Panel C, Figure 4 Panels A, B and C, Figure 5 Panel A, and Figure 7 Panels B and C to new Table 1.

3) The streaming defect is a bit puzzling. Other Dictyostelium mutants with similarly reduced speeds do have streaming defects, such as the MyoIB mutant, that but they are mainly delayed by several hours and not incapable of streaming as is shown here. What can account for the observed phenotype here? Also, in spite of a lack of streaming the mutants appear to form small aggregates - do they go any further in the developmental cycle to form any type of multicellular structure?

We thank Reviewer 3 for their comment, which is similar to a comment made by Reviewer 2 regarding Figure 6 and 7. Our response to Reviewer 2 regarding the streaming/motility/polarity data was: "We thank the reviewer for their comments, which we agree raise significant questions regarding the underlying cause of the fairly severe defect in streaming exhibited by CARMIL-GAP null cells. The fact that this defect is not rescued by complementation of null cells with a version of CARMIL-GAP containing a single point mutation that largely abrogates its GAP activity puts the focus squarely on this activity. While we interpreted the streaming phenotype solely in the context of the GAP-domain dependent regulation of Rac1a (and hence actin assembly/cell migration), we agree that this domain could be also be regulating GTPases that are required for the progression of *Dictyostelium's* developmental program. This possibility remains despite the fact that ripple-stage null cells express near normal levels of CAR1 (new Figure S6). To acknowledge the reviewers important point, we have added the following three sentences at the end of the first paragraph in the Discussion: "It is also important to note that while the discussion below focuses on the role of CARMIL-GAP's GAP domain in regulating Rac1a, we cannot exclude the possibility that this domain regulates additional Rho-related GTPases (e.g. RacE), and that their miss regulation contributes to the defects in actin- dependent processes exhibited by CARMIL-GAP null cells. In a similar vein, we cannot exclude the possibility that the profound defect in streaming exhibited by cells lacking CARMIL-GAP's GAP activity is due at least in part to the miss regulation of GTPases required for progression of *Dictyostelium's* developmental program. The pronounced defect in phagocytosis exhibited by null cells cannot be attributed, however, to defects in this developmental program." Finally, we did not examine CARMIL-GAP mutants on black filters beyond ripple stage so we cannot weigh in on possible/likely defects in their multicellular development.

4) CARMIL-GAP appears to be associated with the cytoskeleton and not exclusively with the membrane (Fig 7D). The authors speculate that the GAP domain could play a role in localization through its interaction with Rac1a. Are the GAP or CPI deletion mutants mislocalized?

We thank Reviewer 3 for raising this issue, which was also raised by Reviewer 1 (see Question 8) and Reviewer 2. Here is our response to Reviewer 2 on this issue: "We thank Reviewer 2 for raising this important issue, which is similar to a concern raised by Reviewer 1. New Figures S4 and S5 show that GFP-CARMIL-GAP, GFP-CARMIL-GAP with the GAP mutation, and GFP- CARMIL-CAP with the CPI mutation all localize to phagocytic cups (Figure S4) and to the leading edge of crawling, ripple-stage cells (Figure S5) in complemented M1 null cells. These results argue that CARMIL-GAP's localization to these sites does not require a functioning GAP domain or a functioning CPI domain. They also argue that the inability of the GAP mutant to rescue either phagocytosis or streaming,

© 2022. Published by The Company of Biologists under the terms of the Creative Commons Attribution License (https://creativecommons.org/licenses/by/4.0/).

and the inability of the CPI mutant to fully rescue these behaviors, cannot be attributed to mis localization. Given this, and given the new western blot data in Figure S3 (see our response to Reviewer 1, Question 7), we conclude that the inability of the GAP mutant to rescue either phagocytosis or streaming, and the inability of the CPI mutant to fully rescue these behaviors, is due to their inability to regulate Rac1a and CP, respectively, rather than to mis localization or insufficient expression."

5) Is CARMIL-GAP unique to Dictyostelium and related species? The authors should briefly comment on this.

We thank Reviewer 3 for this request. In the revised legend to Figure S1 we have included the following statement: "Finally, searches of the mouse genome using two regions of sequence that are highly conserved between vertebrate CARMIL-1, CARMIL-2 and CARMIL-3 did not identify any CARMIL-like proteins that contain a GAP domain, arguing that the regulation of Rho-related GTPases by vertebrate CARMIL proteins (Stark et al., 2017) is restricted to regulation in trans."

6) The statistical analysis is said to be described in an earlier paper, Jung et al, 2016 (PNAS vol 113 - the citation is incomplete) yet that paper does not present any information on statistical analysis in the Methods section. Perhaps one can decipher what was done by going through the paper and figures, but this should not be necessary. It is highly preferable that the statistical analysis be described in the Methods. The number of samples and biological replicates should be reported.

We thank Reviewer 3 for raising this issue and have now included how we performed statistical analyses in the revised Methods. We have also fixed the issue with this citation. Thank you.

Minor points -

a) The gene encoding CARMIL-GAP is currently named gacW (GTPase activator for racC) in Dictybase. The authors should be encouraged to propose a more appropriate gene name to the Dictybase curators.

We thank Reviewer 3 for this suggestion and have contacted the curators of dictyBase to suggest a name change. Of note, the name in Uniprot cannot be changed until our paper is published.

b) There is a straight line at the end of the box diagram of CARMIL-GAP in Fig. 1A. Please describe what this indicates.

The sequence at the C-terminus of CARMIL-GAP that is indicated in the cartoon by the straight line is not present in CARMIL. We thank Reviewer 3 for requesting this clarification, which is now included in the legend to Figure 1.

c) CARMIL was originally identified as a Myosin 1 binding protein, does CARMIL-GAP have a binding site for the SH3 domain of Myosin 1 similar to CARMIL?

We thank Reviewer 3 for this suggestion and searched the proline-rich domain of CARMIL-GAP for the presence of a PXXP-based binding site for the SH3 domain of myosin 1 like the one we identified previously in CARMIL (Jung et al., 2001). The legend to Figure S1 now contains the following statement: "The sequence within the proline-rich domain of CARMIL-GAP that is underlined (residues 920 to 923) marks what may be a PXXP-based binding site for the SH3 domain of myosin 1 (Jung et al. , 2001), although we did not investigate their possible interaction."

d) When describing the complementation experiments that authors say that they 're-express' a GFP fusion of CARMIL-GAP. Strictly speaking they are introducing a GFP- CARMIL-GAP to rescue the null defect.

We thank the reviewer and have changed the manuscript accordingly. For example, the sentence in the Abstract "Importantly, these defects are fully rescued by the re-expression of CARMIL-GAP" was changed to "Importantly, these defects are fully rescued by expressing GFP- tagged CARMIL-GAP in CARMIL-GAP null cells".

© 2022. Published by The Company of Biologists under the terms of the Creative Commons Attribution License (https://creativecommons.org/licenses/by/4.0/).

e) The images of streaming are quite dark. Is it possible to make them a bit lighter to see the whole field a bit better?

We thank the reviewer for their comment and agree that the aesthetics of the images in Figure 7 are not ideal. We did make the images a bit lighter, which we agree helps. While the variations in shading largely remain, we hope the reviewer agrees with us that the images do support the conclusions we reached regarding the role of CARMIL-GAP in steaming, and the contributions that the GAP and CPI domains make to this function.

f) The Discussion states that "Rac1a is expressed at vastly higher levels than Rab1b and Rac1c" and cites Fey et al 2013 (a Methods paper?). The basis for this statement should be explained, presumably the authors are referring to RNA expression levels.

We thank Reviewer 3 for raising this issue and agree that this important point requires further clarification. This statement has its origins in emails with Robert Insall, who we contacted for guidance on Rac1 expression levels. He pasted in his email response a plot of relative RNA levels for Rac1a, Rac1b and Rac1c in vegetative cells and cell undergoing development that he got from dictyExpress. At all stages, the level of Rac1a RNA greatly exceeds that of both Rac1b and Rac1c RNA (by ~40-fold in vegetative cells and, depending on the developmental stage, by ~20- fold to >300-fold). We changed the citation from Fey et al, which introduced dictyBase, to Stajdohar et al, which introduced dictyExpress. In the revised text we indicate that the statement above is based on RNA expression levels measured in both vegetative and developing cells that is available on dictyExpress.

Second decision letter

MS ID#: JOCES/2021/258704

MS TITLE: Dual regulation of the actin cytoskeleton by CARMIL-GAP

AUTHORS: Goeh Jung, Miao Pan, Christopher J. Alexander, Tian Jin, and John A. Hammer
ARTICLE TYPE: Research Article

Sorry it's taken longer than I would have liked but we have now reached a decision on the above manuscript.

To see the reviewers' reports and a copy of this decision letter, please go to: https://submit-jcs.biologists.organd click on the 'Manuscripts with Decisions' queue in the Author Area. (Corresponding author only has access to reviews.)

As you will see, three reviewers gave favourable reports but raised some minor points that will require amendments to your manuscript. I hope that you will be able to carry these out because I will then be able to accept your paper.

Please ensure that you clearly highlight all changes made in the revised manuscript. Please avoid using 'Tracked changes' in Word files as these are lost in PDF conversion.

I should be grateful if you would also provide a point-by-point response detailing how you have dealt with the points raised by the reviewers in the 'Response to Reviewers' box. Please attend to all of the reviewers' comments. If you do not agree with any of their criticisms or suggestions please explain clearly why this is so.

Reviewer 1

Advance summary and potential significance to field

The authors have adequately address all of my comments and concerns.

© 2022. Published by The Company of Biologists under the terms of the Creative Commons Attribution License (https://creativecommons.org/licenses/by/4.0/).

Comments for the author

The authors have revised their manuscript according to my recommendations and those of two other reviewers.

Reviewer 2

Advance summary and potential significance to field

The authors have done a good job at responding to all the previous comments and I feel the paper is almost ready.

Comments for the author

However the way they have presented some of the data with both cell, and experiment level data (e.g. Fig 3C and 4C) remains ambiguous and needs clarification in the legends. Whilst they now add experimental -level averages, it it unclear whether the error bars and statistics are calculated on this or the cell-level datapoints. It is basic good science to explain this clearly and so I recommend this is clarified prior to publication.

Reviewer 3

Advance summary and potential significance to field

see comments in original review

Comments for the author

The reviewers have satisfactorily addressed the comments in the original review.
There are two minor issues outstanding -

1) One question in the original review was whether the CARMIL-GAP mutant can develop beyond the formation of small aggregates (these are seen in the streaming assay, submerged culture). The authors state that they did not examine the mutants on black pads beyond the ripple stage so they cannot comment on this. This response is a bit puzzling - one does not need to assess development only on black pads, the authors plated their mutant on bacterial plates where one can see the presence of fruiting bodies, or not if the mutants cannot progress through the developmental program. The question here is simply whether or not they observed fruiting bodies or some type of abnormal multicellular structure under starvation conditions when the mutants were on bacterial plates.

2) The authors mention blotting samples from cells at the ripple stage (top of pg 10) before explaining to the reader what the ripple stage is.

Second revision

Author response to reviewers' comments

Reviewer 1 Advance Summary and Potential Significance to Field:
The authors have adequately address all of my comments and concerns.

Reviewer 1 Comments for the Author:
The authors have revised their manuscript according to my recommendations and those of two other reviewers.

© 2022. Published by The Company of Biologists under the terms of the Creative Commons Attribution License (https://creativecommons.org/licenses/by/4.0/).

Reviewer 2 Advance Summary and Potential Significance to Field:
The authors have done a good job at responding to all the previous comments and I feel the paper is almost ready.

Reviewer 2 Comments for the Author:
However the way they have presented some of the data with both cell, and experiment level data (e.g. Fig 3C and 4C) remains ambiguous and needs clarification in the legends. Whilst they now add experimental -level averages, it it unclear whether the error bars and statistics are calculated on this or the cell-level datapoints. It is basic good science to explain this clearly and so I recommend this is clarified prior to publication.

I thank the reviewer for pointing out this ambiguity and have now clarified in the legends to Figures 3C, 4C, 7B and 7C that the error bars and statistics are calculated on the cell-level data points.

Reviewer 3 Advance Summary and Potential Significance to Field:
see comments in original review

Reviewer 3 Comments for the Author:
The reviewers have satisfactorily addressed the comments in the original review. There are two minor issues outstanding -

1) One question in the original review was whether the CARMIL-GAP mutant can develop beyond the formation of small aggregates (these are seen in the streaming assay, submerged culture). The authors state that they did not examine the mutants on black pads beyond the ripple stage so they cannot comment on this. This response is a bit puzzling - one does not need to assess development only on black pads, the authors plated their mutant on bacterial plates where one can see the presence of fruiting bodies, or not if the mutants cannot progress through the developmental program. The question here is simply whether or not they observed fruiting bodies or some type of abnormal multicellular structure under starvation conditions when the mutants were on bacterial plates.

I thank the reviewer for pointing this out. Unfortunately, I cannot draw firm conclusions regarding possible defects in the multicellular development of the CARMIL-GAP mutants from the photographs of the plaque assays (they are low mag and were taken from above rather than from the side). I realize that under normal circumstances doing an additional experiment to clarify this point is not hard. In this case, however, it is as the first author retired in November of 2020 and can no longer come to the lab due to health issues. Moreover, he discarded most of his reagents after completing the essential revisions in the summer of 2021. Given this, given that multicellular development is not the focus of this study, and given that addressing this question was not one of the essential revisions enumerated by the monitoring editor, I ask that we please be allowed to proceed with publishing this study in its current form.

2) The authors mention blotting samples from cells at the ripple stage (top of pg 10) before explaining to the reader what the ripple stage is.

I thank the reviewer for this pointing out and now include a brief description of ripple stage at that point in the manuscript: “(the first observable change for cells seeded at high density on black filters in the absence of nutrients; see below and Katoh-Kurasawa et al, 2021)”. This 2021 paper focuses on the transcriptional milestones in Dictyostelium development, including those that occur at ripple stage.

© 2022. Published by The Company of Biologists under the terms of the Creative Commons Attribution License (https://creativecommons.org/licenses/by/4.0/).

<u>Third decision letter</u>

MS ID#: JOCES/2021/258704

MS TITLE: Dual regulation of the actin cytoskeleton by CARMIL-GAP

AUTHORS: Goeh Jung, Miao Pan, Christopher J. Alexander, Tian Jin, and John A. Hammer
ARTICLE TYPE: Research Article

I am happy to tell you that your manuscript has been accepted for publication in Journal of Cell Science, pending standard ethics checks.

© 2022. Published by The Company of Biologists under the terms of the Creative Commons Attribution License (https://creativecommons.org/licenses/by/4.0/).
